# Lattice Anchoring Stabilizes α-FAPbI_3_ Perovskite for High-Performance X-Ray Detectors

**DOI:** 10.1007/s40820-025-01856-4

**Published:** 2025-07-29

**Authors:** Yu-Hua Huang, Su-Yan Zou, Cong-Yi Sheng, Yu-Chuang Fang, Xu-Dong Wang, Wei Wei, Wen-Guang Li, Dai-Bin Kuang

**Affiliations:** Key Laboratory of Bioinorganic and Synthetic Chemistry of Ministry of Education, LIFM, GBRCE for Functional Molecular Engineering, School of Chemistry, IGCME, Sun Yat-Sen University, Guangzhou, 510275 People’s Republic of China

**Keywords:** α-FAPbI_3_ perovskite, Conjugated organic cation, Lattice anchoring, Phase stability, X-ray detectors

## Abstract

Formamidinium lead iodide (FAPbI_3_) perovskite exhibits an impressive X-ray absorption coefficient and a large carrier mobility-lifetime product (µτ), making it as a highly promising candidate for X-ray detection application. However, the presence of larger FA^+^ cation induces to an expansion of the Pb-I octahedral framework, which unfortunately affects both the stability and charge carrier mobility of the corresponding devices. To address this challenge, we develop a novel low-dimensional (HtrzT)PbI_3_ perovskite featuring a conjugated organic cation (1H-1,2,4-Triazole-3-thiol, HtrzT^+^) which matches well with the α-FAPbI_3_ lattices in two-dimensional plane. Benefiting from the matched lattice between (HtrzT)PbI_3_ and α-FAPbI_3_, the anchored lattice enhances the Pb-I bond strength and effectively mitigates the inherent tensile strain of the α-FAPbI_3_ crystal lattice. The X-ray detector based on (HtrzT)PbI_3_(1.0)/FAPbI_3_ device achieves a remarkable sensitivity up to 1.83 × 10^5^ μC Gy_air_^−1^ cm^−2^, along with a low detection limit of 27.6 nGy_air_ s^−1^, attributed to the release of residual stress, and the enhancement in carrier mobility-lifetime product. Furthermore, the detector exhibits outstanding stability under X-ray irradiation with tolerating doses equivalent to nearly 1.17 × 10^6^ chest imaging doses.

**Supplementary Information:**

The online version contains supplementary material available at 10.1007/s40820-025-01856-4.

## Highlights

- A lattice-anchoring strategy using low-dimensional perovskite addresses structural instability in α-formamidinium lead iodide (FAPbI_3_) by matching crystal lattice, mitigating residual stress and tensile strain.
- Enhanced Pb-I bonding strength and reduced lattice strain improve structural stability and carrier mobility-lifetime product, enabling efficient charge transport.
- Optimized X-ray detectors achieve high sensitivity (1.83 × 10^5^ μC Gy_air_^–1^ cm^–2^), low detection limit (27.6 nGy_air_ s^–1^), and stable performance under prolonged irradiation.

**Supplementary Information:**

The online version contains supplementary material available at 10.1007/s40820-025-01856-4.

## Graphical abstract

**Figure:**
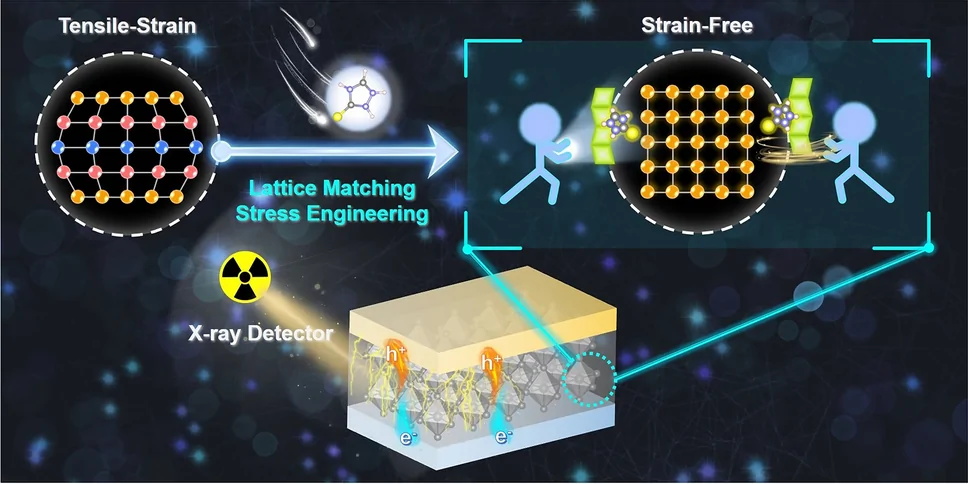

## Introduction

X-ray detectors play a crucial role in various domains such as medical imaging, industrial nondestructive testing, and security inspections, enabling the observation of internal morphological characteristics of objects [1]. However, several potential applications of commercial detectors are constrained by their own drawbacks such as their high cost, limited spatial resolution, or small detection area [2]. For instance, the α-Se detector demonstrates excellent performance in soft X-ray detection; however, its effectiveness significantly diminishes for hard X-ray detection due to limited X-ray attenuation capacity above 50 keV [3]. The limitations of CdZnTe (CZT) X-ray detector, such as its small area, high cost, and the requirement for high-voltage operation, pose challenges to practical applications [4].

Halide perovskites hold great promise for achieving high-performance X-ray direct detectors [5–7], due to the advantages such as low cost [1], high X-ray absorption coefficient [8, 9], and excellent charge transport ability [10, 11]. Currently, significant research achievements have been obtained for methylamine (MA)-based perovskite X-ray detectors [12–14]. However, the inherent volatility of MA poses a significant challenge to the long-term stability of corresponding devices [15]. In contrast, formamidinium (FA)-based perovskite possesses merits such as a small ionization energy and high thermal stability [16, 17], rendering it a promising material for optoelectronic devices. Nevertheless, the large size of FA^+^ ions result in a considerable tensile stress of lattice in the corresponding perovskite, rendering the black cubic phase (α-FAPbI_3_) prone to phase transition to the non-photoactive yellow hexagonal phase (δ-FAPbI_3_) at room temperature [18, 19]. Therefore, the tendency of phase transition for α-FAPbI_3_ constitutes a crucial issue influencing the stability of perovskite devices.

Chemical engineering is a commonly employed strategy to enhance the phase stability of FA-perovskites by introducing small cations (such as MA^+^, Cs^+^, and Rb^+^) [20] or large long-chain organic cations [21–29]. However, the common small cations partially substituting FA^+^ can result in an increased bandgap and ionization energy, leading to reduced carrier generation under X-ray excitation [22, 30, 31]. Additionally, low-dimensional (LD) perovskites can facilitate the nucleation of α-FAPbI_3_ at the LD perovskite/FAPbI_3_ coherent interface. However, the low conductivity of long-chain organic cation-based LD perovskite derivatives along grain boundaries or surfaces hinder efficient carrier transport [32]. Therefore, it is necessary to explore more superior strategies to improve the phase stability of FA-based perovskites.

Compared to long-chain organic cation-based LD perovskites, the introduction of semiconducting conjugated organic cations into perovskites can enhance the transport of charge carriers [9, 33–36]. Moreover, when conjugated organic cations containing electron-donating groups are present, they are capable of coordinating with coordinatively unsaturated lead in perovskites [37–41] and can effectively passivate defects. Therefore, constructing a lattice-matched LD perovskite/FAPbI_3_ coherent interface by conjugated organic cation-based LD perovskite is a promising choice for mitigating the lattice expansion of the Pb-I octahedral framework in α-FAPbI_3_ perovskite.

In this study, a conjugated organic cation (1H-1,2,4-Triazole-3-thiol, HtrzT^+^) formed a trace of LD (HtrzT)PbI_3_ perovskite at grain boundaries of FAPbI_3_ perovskite. The (HtrzT)PbI_3_ exhibits an exquisite lattice matching with the α-FAPbI_3_ lattice in the two-dimensional plane, thereby effectively reducing the lattice strain of α-FAPbI_3_. The reduced lattice stress enables a highly stable octahedral framework, resulting in a lowered formation energy and inhibition of phase transition. Subsequently, a high-quality α-FAPbI_3_ thick film with large grain sizes and low defect density was prepared using a blading-coating and soft hot-pressing method. Consequently, the X-ray detector based on (HtrzT)PbI_3_(1.0)/FAPbI_3_ demonstrates a high sensitivity (1.83 × 10^5^ μC Gy_air_^−1^ cm^−2^) and a low detection limit (27.6 nGy_air_ s^−1^), attributing to its prolonged carrier lifetime and enhanced carrier transport performance. Additionally, it exhibits excellent stability when exposed to X-ray irradiation (over 117 Gy_air_).

## Experimental Section

### Materials

Formamidine acetate (FAAc, 99%), hypophosphorous acid (H_3_PO_2_, 50 wt% in H_2_O), 3-aminopropyl-triethoxysilane (APTES, 99%), 2-methoxyethanol (2-ME, 99.8%) and 1-methyl-2-pyrrolidinone (NMP, 99.5%) were purchased from Aladdin. Lead iodide (PbI_2_, 98%) and hydroiodic acid (HI, 55–58 wt%) were purchased from Macklin. 1H-1,2,4-triazole-3-thiol (HtrzT, 95%) was purchased from Bidepharm. Chlorobenzene (CB, 99.5%) and isopropyl alcohol (IPA, 99.7%) were purchased from Guangzhou Chemical Reagent. All chemicals were used as received without any further purification.

### Synthesis of (HtrzT)PbI_3_ Single Crystals

9 mmol HtrzT and 8 mmol PbI_2_ were added to a 4 mL HI and 0.2 mL H_3_PO_2_ solution and stirred at 70 °C until dissolved. After the solution cooled to room temperature naturally, yellow needlelike (HtrzT)PbI_3_ crystals were obtained.

### Synthesis of α-FAPbI_3_ and (HtrzT)PbI_3_/FAPbI_3_ Microcrystals

Organic precursor solution was prepared by dissolving HtrzT and FAAc (mole ratios of HtrzT:FA were 0:1, 0.5:1, 1:1 and 1.5:1) in HI and H_3_PO_2_ solution (25:1, v:v), according to Table S1. And the transparent solution was obtained through stirring at 70 °C. For PbI_2_ precursor solution, 5 mmol PbI_2_ was dissolved in 4 mL HI and 160 μL H_3_PO_2_ solution at room temperature and stirred until a transparent solution was obtained. The precipitated powders were synthesized by mixing organic and PbI_2_ precursor solutions and separated from the solution at room temperature. The filtered powders were heated on a hot plate at 150 °C for 1 h in N_2_ atmosphere.

### Device Fabrication

The FTO glasses were initially cleaned using deionized water, followed by a mixture of acetone and isopropyl alcohol (1:1, v:v), and ethanol through ultrasonic treatment, and then dried for subsequent use. The FTO glasses were treated in a UV-ozone machine for 20 min, followed by immersion in a 5 vol% APTES chlorobenzene solution at 60 °C for 12 h. Then, the substrates were placed into isopropyl alcohol for ultrasonic cleaning for 2 min, followed by drying with nitrogen gas for subsequent use. 1 perovskite microcrystals, 400 μL 2-ME and 40 μL NMP were put into a mortar and ground for ~ 1 min to obtain the perovskite slurry. The slurry was applied to the substrate using a blade-coating method. The coated substrate was cured on a hot plate at 150 °C for 1 h. After that, the perovskite thick films were transferred to a heat press machine and subjected to pressing at 100 °C and 10 MPa for 1 h, followed by cooling to room temperature before removing out. Finally, ~ 100-nm Au electrode was sputtered on the top surface of the perovskite films using a metal mask.

### Characterizations

X-ray diffraction (XRD) patterns of the samples were collected by a Miniflex600 X-ray diffractometer using Cu K_α_ radiation (*λ* = 1.5418 Å). The microstructure of the samples was observed by high-resolution emission scanning electron microscope (ZEISS, Gemini 500) and spherical aberration corrected transmission electron microscope (JEM-ARM200P). The Fourier transform infrared (FTIR) spectra were recorded by a PerkinElmer Frontier spectrophotometer. Raman measurements on a Renishaw InVia Reflex confocal Raman spectrometer with a laser (*λ* = 532 nm). The X-ray photoelectron spectra (XPS) were performed on a photoelectron spectrometer (ESCALAB 250, Thermo Fisher Scientific). Differential scanning calorimetry (DSC) analysis was carried out on Netzsch DSC 214 instrument. The absorption spectra of the films were recorded on a Shimadzu UV-3600 UV–vis spectrometer with an integrated sphere. The steady-state and transient PL spectra of the samples were measured using a combined fluorescence spectrometer (Edinburgh, FLS1000) with an excitation wavelength of 450 nm. The PL mappings was measured by Raman imaging microscope (WITec alpha300R). Atomic force microscopy (AFM) and Kelvin probe force microscope (KPFM) measurements were performed on a Bruker Dimension Fast Scan AFM system. For the photoconductivity measurement, the device was illuminated with a 405-nm laser, and the response photocurrent was recorded using an electrochemical workstation. The noise measurement was conducted using an FS-Pro semiconductor parameter analyzer.

### Grazing-Incidence X-Ray Diffraction (GIXRD) Measurements

The GIXRD measurements were conducted at the X-ray diffractometer (Panalytical, Empyrean) in conjunction with the Williamson–Hall plot [42, 43]:

1 $$\frac{\beta \mathrm{cos}\theta }{\lambda }=\frac{K}{D}+2\varepsilon \left(\frac{2\mathrm{sin}\theta }{\lambda }\right)$$

where *β* is the full width at half maximum, *θ* is the Bragg angle, *λ* is the wavelength of the X-ray, *K* is the Scherrer constant, *D* is the crystallite size, and *ε* is the micro-strain.

### Space-­Charge-­Limited Current Measurement

The trap density (*n*_*t*_) was derived from the trap­filled limit voltage (*V*_TFL_) of the linear Ohmic region, utilizing the subsequent Eq. 2 [10]:

2 $${V}_{\mathrm{TFL}}=\frac{q{n}_{t}{L}^{2}}{2\varepsilon {\varepsilon }_{0}}$$

where *q* is the elementary charge (1.6 × 10^–19^ C), *L* is the thickness of the films, *ε* is relative dielectric constant, and *ε*_*0*_ is the vacuum permittivity (8.854 × 10^–14^ F cm^−1^). Concurrently, the carrier mobility (*μ*) was evaluated by fitting with Mott–Gurney’s equation at the Child’s region [10]:

3 $${J}_{D}=\frac{9\varepsilon {\varepsilon }_{0}\mu {V}_{b}^{2}}{8{L}^{3}}$$

where *J*_*D*_ and *V*_*b*_ are the current density and applied voltage, respectively.

### Photoconductivity Measurement

The carrier mobility-lifetime (*μτ*) products were carried out using the photoconductivity method extended from the fitting of the Hecht equation [44]:

4 $$I=\frac{{I}_{0}\mu \tau V}{{L}^{2}}\frac{1-\mathrm{exp}(-\frac{{L}^{2}}{\mu \tau V})}{1+\frac{Ls}{V\mu }}$$

where *I* is the photocurrent, *I*_0_ is the saturated photocurrent, *V* is the bias voltage, and *s* is the surface recombination velocity.

### Time-of-Flight (ToF) Measurement

The ToF method was utilized to measure carrier mobility by illuminating with 405-nm laser pulses and calculating from the Eq. 5 [44]:

5 $${\tau }_{t}=\frac{{L}^{2}}{\mu V}$$

where *τ*_*t*_ is the transit time of the charge carriers, *L* is the thickness of the films, *μ* is the charge carrier mobility, and *V* is the bias voltage.

### Transient Absorption (TA) Spectroscopy Characterizations

The TA spectra were measured on a Helios transient absorption spectrometer pump-probe setup (Ultrafast Systems, LLC) in reflection mode over the visible and near-infrared region from 650 to 840 nm. The pump beam at 350 nm was generated by the second-harmonic generation of 800 nm fundamental pulses (100 fs, 1 kHz repetition rate, Astrella-Tunable-V-F-1 k, Coherent) from an optical parametric amplifier (OPerA Solo, Coherent).

### X-Ray Detector Characterizations

A Mini-X2 X-ray tube (Ag target) from Amptek, Inc. was served as the X-ray source. The tube voltage was fixed at 40 kV, while the tube current could be adjusted between 10 and 100 µA to regulate the dose rate. The dose rate was calibrated using an ion chamber dosimeter (10X6-6 M, Radcal). The current–time curves of the devices response to X-ray irradiation were recorded in a metal vacuum chamber with a beryllium window (1 Torr). For the X-ray imaging measurements, both the X-ray source and detector were fixed in position, while the object placed between them was moved utilizing a x–y scanning platform. The signal-to-noise ratios (SNR) of the devices were calculated through the following equations [45]:

6 $${I}_{\mathrm{signal}}={\overline{I} }_{\mathrm{photo}}-{\overline{I} }_{\mathrm{dark}}$$

7 $${I}_{\mathrm{noise}}=\sqrt{\frac{1}{N}\sum_{i}^{N}{\left({I}_{i}-{\overline{I} }_{\mathrm{photo}}\right)}^{2}}$$

8 $${\mathrm{SNR}} = I_{{{\mathrm{signal}}}} /I_{{{\mathrm{noise}}}}$$

where *I*_signal_ is signal current, *I̅*_photo_ is average photocurrent, *I̅*_dark_ is average dark current, and *I*_noise_ is noise current.

Theoretical sensitivity (*S*_0_) can be estimated using the following formula, assuming 100% photon-to-photocurrent conversion efficiency [14, 46]:

9 $${S}_{0}=\frac{\frac{\varnothing }{X}E\beta }{{W}_{\pm }}e\eta$$

where *ϕ*/*Χ* denotes the number of photons per unit of exposure, *E* is the mean energy of X-ray photons, and *β* is the energy attenuation efficiency. The mean ionization energy required to generate an electron–hole pair (*W*_±_) is given by *W*_±_ = 2*E*_g_ + 1.43 [47], where *E*_g_ is the bandgap energy. Additionally, *e* is the element electron charge, and *η* is the charge collection efficiency. Gain factor (*G*) is defined as *G* = *S*/*S*_0_, where *S* represents the experimental sensitivity.

### Computational Details

All calculations were carried out by means of the density functional theory within the projector augmented plane-wave (PAW) method, as implemented in the Vienna ab initio simulation package [48]. The exchange–correlation potential was depicted through the generalized gradient approximation (GGA) put forward by Perdew, Burke, and Ernzerhof [49]. Long-range van der Waals interactions were taken into consideration by means of the DFT-D3 approach [50]. A plane-wave cutoff energy of 400 eV was employed. Convergence in the iterative solution of the Kohn–Sham equation was attained with an energy criterion of 10^–5^ eV. A vacuum layer of 25 Å was utilized to avoid artificial interactions among periodic images. Structural optimizations were carried on until the residual forces on all atoms dropped beneath 0.03 eV Å^−1^. The formation energy of HtrzT^+^ on the surfaces of FAPbI_3_ perovskite was calculated using the equation Δ*E*_form_ = *E*_total_ - (*E*_surface_ + *E*_HtrzT_^+^), while the formation energy of the perovskites with different phases was calculated using the formula Δ*E*_form_ = *E*_total_ - (*n* × *E*PbI_2_ + *m* × *E*_FAI_ - *l* × μ_I_ + *E*_HtrzT_^+^), where the *E*_total_ and *E*_surface_ are the energy of the perovskite with and without HtrzT^+^. *E*_HtrzT_^+^, *E*PbI_2_, *E*_FAI_ are the energies of HtrzT^+^, PbI_2_, and FAI, respectively. *n*, *m*, and *l* are numbers of the PbI_2_, FAI, and vacancy of I in the calculated perovskite.

## Results and Discussion

### Phase Composition Analysis of (HtrzT)PbI_3_/FAPbI_3_ Hybrid Perovskites

The FAPbI_3_ and (HtrzT)PbI_3_/FAPbI_3_ hybrid microcrystals were synthesized in HI solutions, where the FAI and PbI_2_ with varying amounts of HtrzT organic ligand were dissolved (Table S1). Here, the mole ratios of HtrzT:FA were 0:1, 0.5:1, 1:1, and 1.5:1, and the corresponding samples were denoted as FAPbI_3_, (HtrzT)PbI_3_(0.5)/FAPbI_3_, (HtrzT)PbI_3_(1.0)/FAPbI_3_, and (HtrzT)PbI_3_(1.5)/FAPbI_3_, where the stoichiometry ratios of HtrzT to FA in the final perovskites were measured to be 0.012:1, 0.024:1, and 0.034:1 by the ^1^H NMR spectrum (Fig. S1), respectively. The (HtrzT)PbI_3_/FAPbI_3_ hybrid microcrystals (ranging from red to yellow) can be transformed into black microcrystals after annealing at 150 °C for 30 min as same as pure FAPbI_3_ (Figs. S2 and S3). The XRD analysis of black microcrystals revealed that the diffraction peaks can be assigned to the characteristic features of the α-FAPbI_3_ phase (Fig. S4a). Normally, pure FAPbI_3_ tends to spontaneously transform from α phase to δ phase due to lattice expansion. Upon introducing (HtrzT)PbI_3_, it is observed that the (002) diffraction peak position at ~ 28° gradually shifted toward higher angles as the content of (HtrzT)PbI_3_ increased, indicating lattice contraction of α-FAPbI_3_ (Fig. S4b).

**Fig. 1:**
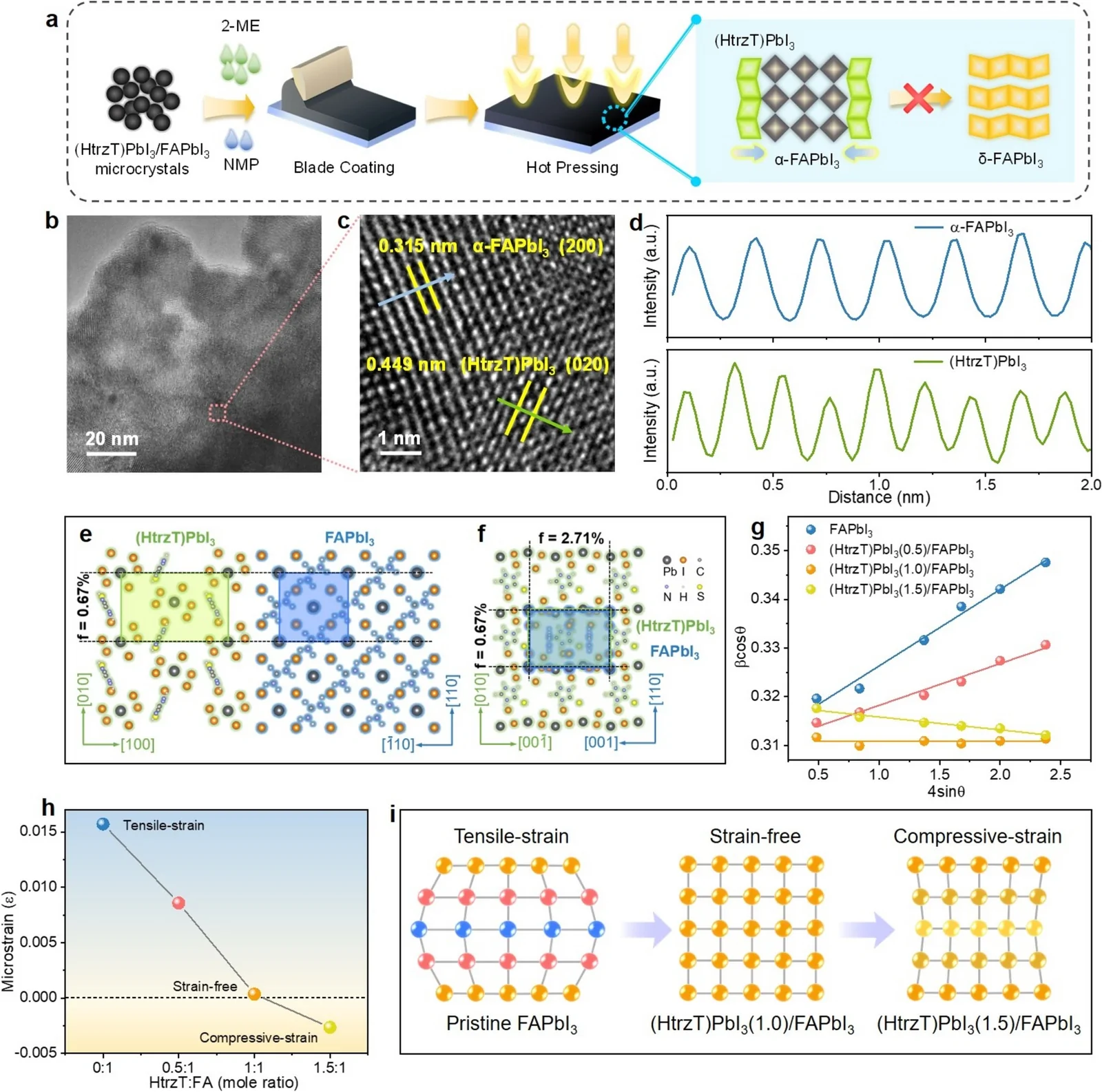
Crystal structure and lattice stress of (HtrzT)PbI_3_/FAPbI_3_ perovskite. **a** Diagram of a blading-coating and hot-pressing method for preparing perovskite thick films. **b** High-resolution transmission electron microscopy (TEM) image of (HtrzT)PbI_3_/FAPbI_3_. **c** Expansion of the pink dashed square in **b** corresponding to the lattice of FAPbI_3_ and (HtrzT)PbI_3_ perovskites, and **d** the corresponding interplanar spacing. **e, f** Illustration of lattice match between (HtrzT)PbI_3_ and FAPbI_3_. **g** Williamson–Hall plots of FAPbI_3_ and (HtrzT)PbI_3_/FAPbI_3_ films. **h** Residual strain calculated in corresponding films. **i** Schematic diagram of lattice structure with/without strain state

The perovskite thick films were prepared by a blading-coating and soft hot-pressing method (Fig. 1a). It is worth noting that the perovskite precursor suspension for blade coating was prepared using a mixture of 2-ME and NMP as solvents. This choice was based on the fact that 2-ME exhibits a weak coordination ability with Pb^2+^, which can form a stable supersaturated perovskite suspension [51]. On the other hand, NMP can coordinate with PbI_2_ to form a PbI_2_·NMP adduct, which is beneficial for the formation of the α-phase FAPbI_3_ [52]. After blading coating and annealing, there are numerous of pores within the resulting black perovskite thick film (Fig. S5a-c). Subsequently, a hot-pressing process (100 °C, 10 MPa, 1 h) can facilitate grains fusion, effectively reducing the number of pores inside the film. As a result, after hot-pressing treatment, the (HtrzT)PbI_3_(1.0)/FAPbI_3_ film exhibited improved surface smoothness, with its thickness being reduced from approximately 160 to 41 μm (Fig. S5d-f). Under the same processing conditions, perovskite films with varying stoichiometric ratios also exhibited comparable thickness values approaching 40 μm after hot-pressing (Fig. S6), thereby confirming the excellent reproducibility of this fabrication process.

Given the limited amount of HtrzT added and the insufficient resolution of XRD phase content analysis, the existence of LD perovskite was confirmed using transmission electron microscopy (TEM) and energy dispersive spectrometer (EDS). The TEM images revealed the formation of a new lattice adjacent to the α-FAPbI_3_ lattice (Fig. 1b, c). The crystallographic analysis reveals that the FFT image (Fig. S7b) in blue-boxed region of Fig. S7a resolves characteristic planes with measured d-spacings of 3.15 and 2.23 Å (interplanar angle = 45°), consistent with the (002) and (0 $\overline{2 }$ 2) planes of cubic phase of α-FAPbI_3_. In contrast, the FFT image in Fig. S7c demonstrates orthorhombic symmetry of (HtrzT)PbI_3_ phase through its (110) and (030) planes (*d* = 7.61/3.03 Å; interplanar angle = 33°). Furthermore, well-defined lattice fringes with lattice spacing of 0.449 nm (Fig. 1d) correspond to the (020) plane for the one-dimensional perovskite of (HtrzT)PbI_3_ (shown in Fig. S8 and Table S2 for crystal structure). It is worth noting that the (HtrzT)PbI_3_ exhibits excellent lattice matching with α-FAPbI_3_. Specifically, the vertical distance between Pb atoms in the (HtrzT)PbI_3_ unit cell closely matches that in the α-FAPbI_3_ unit cell in the (HtrzT)PbI_3_[010]∥α-FAPbI_3_[110] direction, with a very low lattice mismatch factor (*f*) of only 0.67% [*f* = (1 - d((HtrzT)PbI_3_)/d(α-FAPbI_3_)), where d is the interatomic spacing] (Fig. 1e). Moreover, 1.5 unit cells of (HtrzT)PbI_3_ can align with two α-FAPbI_3_ unit cells in the (HtrzT)PbI_3_[001]∥α-FAPbI_3_[001] direction, resulting in a lattice mismatch of 2.71% (Fig. 1f). Matching lattice for the alignment of crystal lattices between a LD perovskite and a three-dimensional (3D) perovskite in two-dimensional orientations, is crucial for minimizing defects of 3D perovskites during the growth process and thereby improving the stability of α-FAPbI_3_ [32, 53–58]. In addition, EDS line-scan analysis further identified distinct S element enrichment at grain boundary, providing direct evidence that (HtrzT)PbI_3_ preferentially localizes along FAPbI_3_ perovskite grain boundaries and surface regions rather than within grain interiors (Fig. S9).

In order to further investigate the effect of matched lattice on lattice strain of perovskite films, the micro-strain (*ε*) was determined by calculating the slope of the fitted Williamson–Hall plot using the grazing-incidence X-ray diffraction (GIXRD) technique (Fig. 1g) [42, 43]. The (HtrzT)PbI_3_/FAPbI_3_ perovskite films display smaller slopes of fitted lines (from 8.57 × 10^–3^ to − 2.65 × 10^–3^) compared to that of the FAPbI_3_ film (1.57 × 10^–2^), indicating that the crystal lattice is compressed. As the content of (HtrzT)PbI_3_ increases, the residual tensile strain in the perovskite crystal structure gradually decreases; in particular, the film of (HtrzT)PbI_3_(1.0)/FAPbI_3_ results in the minimal strain (3.50 × 10^–4^), thereby effectively mitigating lattice strain within the perovskite crystal structure (Fig. 1h and i). Further increasing the (HtrzT)PbI_3_ content, the (HtrzT)PbI_3_(1.5)/FAPbI_3_ film even exhibits compressive strain. This finding highlights a significant effect on strain regulation, potentially attributed to alignment matching between (HtrzT)PbI_3_ lattice and FAPbI_3_ lattice. Consequently, the lattice expansion of FAPbI_3_ crystal structure is effectively suppressed, leading to a reduction in lattice stress and minimizing lattice distortion. This phenomenon is expected to significantly enhance the stability of α-FAPbI_3_ phase.

### Coordination and Phase Transition Characteristics

**Fig. 2:**
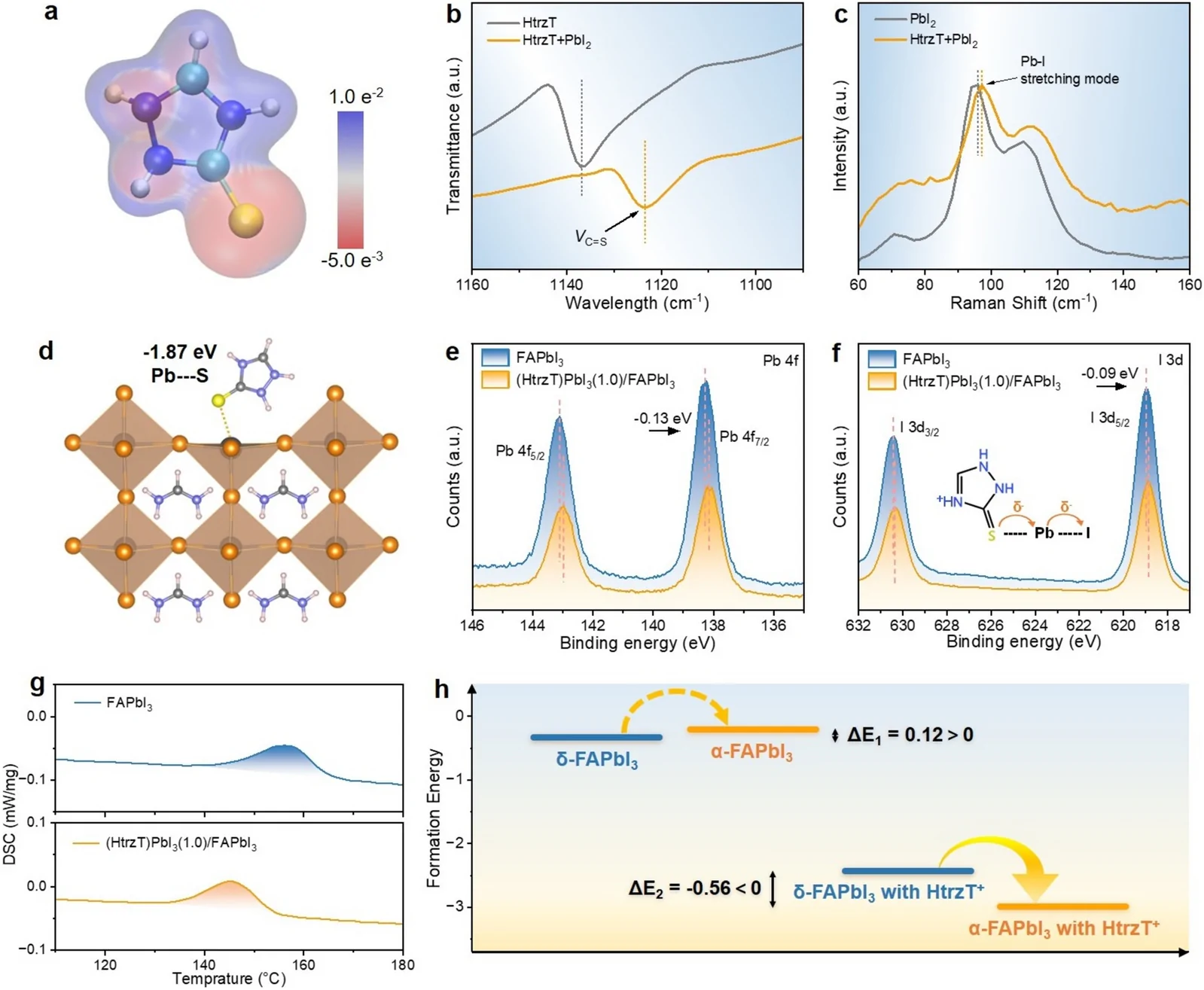
Coordination with HtrzT and phase transition in FA-based perovskite. **a** Electrostatic surface potential (ESP) image of HtrzT^+^. **b** Fourier transform infrared (FTIR) spectra and **c** Raman spectra of HtrzT and HtrzT-PbI_2_ complex. **d** Diagrams of calculated binding free energy of FAPbI_3_ and HtrzT^+^. XPS spectra of FAPbI_3_ and (HtrzT)PbI_3_(1.0)/FAPbI_3_ perovskite: **e** Pb and **f** I. **g** Differential scanning calorimetry (DSC) of FAPbI_3_ and (HtrzT)PbI_3_(1.0)/FAPbI_3_ perovskite. **h** DFT calculations of the formation energy for perovskite

Generally, polar molecules and functional groups with lone pairs of electrons are conducive to defect passivation for perovskite films [59, 60]. To gain insight into the impact of the HtrzT^+^ organic cation on the crystallization process of α-FAPbI_3_, density functional theory (DFT) calculations were performed. The electrostatic potential (ESP, Fig. 2a) results demonstrate that the majority of negative charges in the HtrzT^+^ are concentrated at the thiol group (= S). This spatial distribution facilitates favorable coordination interactions between HtrzT^+^ and the uncoordinated Pb ions present in the α-FAPbI_3_ perovskite. Such strong chemical interaction has been further substantiated through Fourier transform infrared spectroscopy (FTIR) and Raman spectroscopy. The FTIR results showed that the stretching vibrations of C=S bonds in HtrzT shift from 1137 to 1123 cm^−1^ after mixed with PbI_2_ (Fig. 2b). Furthermore, upon the introduction of HtrzT, the stretching-vibration peak of Pb-I bond in PbI_2_ demonstrates a shift from 96.0 to 97.2 cm^−1^ as observed in Raman spectroscopy (Fig. 2c), confirming the interaction between HtrzT and Pb-I framework. Additionally, the calculated binding free energy between Pb atom of α-FAPbI_3_ and the S atom of the HtrzT^+^ is − 1.87 eV, further suggesting that dissociative HtrzT^+^ at the grain boundaries trends to coordinate with α-FAPbI_3_ (Fig. 2d). The observed shift of Pb 4*f* and I 3*d* peaks toward lower binding energy in the (HtrzT)PbI_3_(1.0)/FAPbI_3_ perovskite can be attributed to the increased electron densities of Pb and I, further confirming these interactions (Fig. 2e, f). The matched lattice and robust interfacial coupling provide opportunities to regulate the thermodynamics of phase transition thermodynamics and crystallization kinetics in α-FAPbI_3_. Differential scanning calorimetry (DSC) analysis confirms that the (HtrzT)PbI_3_(1.0)/FAPbI_3_ composite demonstrates a lower phase transition temperature (145 °C) compared to pristine FAPbI_3_ (156 °C, Fig. 2g), both below their decomposition temperature (331 °C, Fig. S10). Additionally, the (HtrzT)PbI_3_(1.0)/FAPbI_3_ composite exhibits a reduced phase transition enthalpy (3.97 vs. 4.81 J g^−1^). These observations can be attributed to variations in perovskite formation energy and energy barriers for phase conversion. The mitigation of tensile strain diminishes the energy barrier for phase transition from δ phase to α phase, whereas the presence of compressive strain within the α phase significantly heightens the energy barrier for its reversion to δ phase, thus profoundly decelerating the transformative process from α phase to δ phase [53]. DFT calculations indicate that the introduction of HtrzT^+^ leads to a lower formation energy for the α-FAPbI_3_ compared to the δ-FAPbI_3_ (Fig. 2h), thereby suppressing the transformation from α-phase to δ-phase. The long-term stability test revealed that (HtrzT)PbI_3_/FAPbI_3_ microcrystals and films maintained the α-FAPbI_3_ phase even after being stored in a nitrogen atmosphere for over 100 days (Figs. S11 and S12). Nevertheless, the XRD characterization revealed the presence of PbI_2_ impurities in the pristine FAPbI_3_ film (Fig. S12a, c). After being stored in a nitrogen atmosphere for 105 days, the intensity diffraction peak of PbI_2_ increased, and a portion of the α phase underwent a phase transformation to the δ phase for pristine FAPbI_3_ film, simultaneously (Fig. S12b). In addition, after 60 h of light-soaking exposure, the (HtrzT)PbI_3_(1.0)/FAPbI_3_ composite maintained superior phase stability, whereas pristine FAPbI_3_ underwent structural decomposition under the same conditions, as evidenced by the appearance of multiple degradation-related diffraction peaks (Fig. S13). The above characterizations indicate that the introduction of (HtrzT)PbI_3_ can significantly enhance the phase stability and photostability of FAPbI_3_.

### Optical Property Characterization

**Fig. 3:**
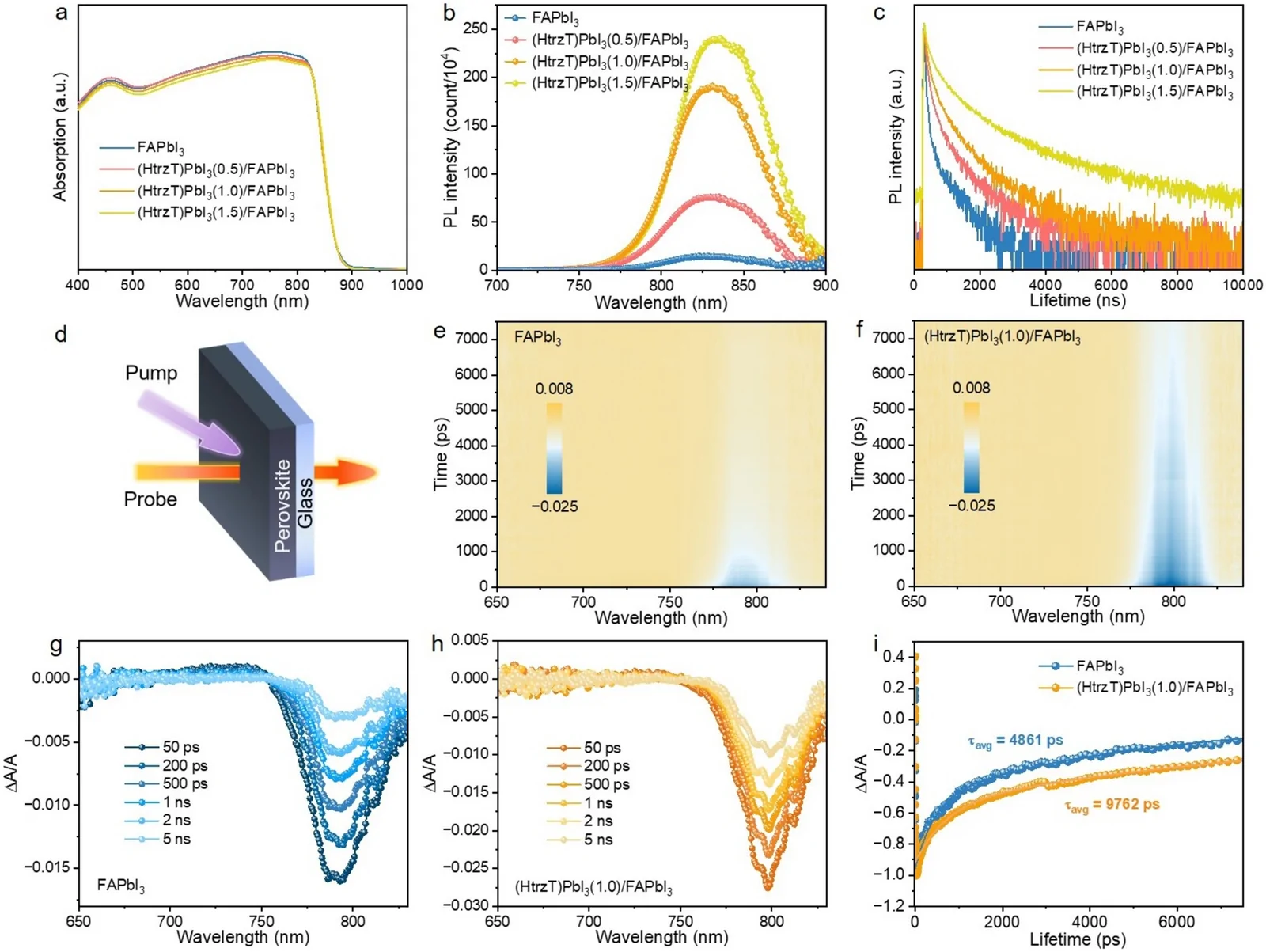
Absorption spectra, photoluminescence properties and transient absorption spectroscopic measurements. **a** Electronic absorption spectra, **b** PL spectra and **c** TRPL spectra of α-FAPbI_3_ and (HtrzT)PbI_3_(1.0)/FAPbI_3_. **d** Schematic of the perovskite film for TA measurement. **e, f** TA spectra pseudocolor images and **g, h** TA spectra recorded at different delay time of FAPbI_3_ and (HtrzT)PbI_3_(1.0)/FAPbI_3_ perovskite films. **i** TA kinetic traces of corresponding perovskite films

The optical properties of perovskite thick films were conducted to investigate the generation, recombination, and dynamics of charge carriers. Absorption spectroscopy characterization (Fig. 3a) reveals that the absorption spectra of (HtrzT)PbI_3_/FAPbI_3_ films incorporating LD perovskite are indistinguishable from that of pure α-FAPbI_3_ film, which can be attributed to the limited content of (HtrzT)PbI_3_ perovskite components. In other words, the similar absorption edges suggest that the trace amount of (HtrzT)PbI_3_ has a negligible effect on the bandgap (1.44 eV) of the α-FAPbI_3_ films, which is conducive to maintaining a low electron–hole pair formation energy. X-ray photoelectron spectroscopy (XPS) analysis reveals that (HtrzT)PbI_3_ has a lower work function than FAPbI_3_ (Fig. S14a-c), indicating electrons transfer from (HtrzT)PbI_3_ to FAPbI_3_ at their interface. Consequently, combination of absorption spectra for (HtrzT)PbI_3_ (Fig. S15) and α-FAPbI_3_ demonstrates the formation of a type-I heterojunction between (HtrzT)PbI_3_ and α-FAPbI_3_ upon reaching equilibrium (Fig. S14d). The steady-state photoluminescence (PL) and time-resolved photoluminescence (TRPL) results reveal that the (HtrzT)PbI_3_/FAPbI_3_ perovskite films exhibit a significantly enhancement in PL intensity (Fig. 3b) and longer PL lifetime (from 137 to 806 ns) as the addition amount of HtrzT increases (Fig. 3c and Table S3). The PL mappings also confirm that the (HtrzT)PbI_3_(1.0)/FAPbI_3_ perovskite film displays enhanced PL intensity compared with the pristine α-FAPbI_3_ film (Fig. S16). This phenomenon can be attributed to the formation of a type-I heterojunction, while the presence of LD (HtrzT)PbI_3_ further reduces the non-radiative recombination associated with defect states in perovskite films.

To deeply explore the influence of (HtrzT)PbI_3_ on the intrinsic charge carrier dynamics in perovskite films, transient absorption (TA) spectroscopy was further carried out upon 350 nm laser excitation (Fig. 3d). Figure 3e, f illustrates the pseudocolor 2D maps of the TA spectra of α-FAPbI_3_ and (HtrzT)PbI_3_(1.0)/FAPbI_3_ perovskite films, respectively, where significant ground-state bleaching (GSB) peaks at approximately 795 nm are observed. Relative to pristine α-FAPbI_3_ film, the (HtrzT)PbI_3_(1.0)/FAPbI_3_ film exhibits a stronger GSB signal. In addition, the intensity of the GSB peak in the (HtrzT)PbI_3_(1.0)/FAPbI_3_ perovskite film decays more slowly at the same delay time compared with the pristine α-FAPbI_3_ film (Fig. 3g, h). The decay kinetics probed above GSB peaks have been further analyzed by a double exponential fitting mode to delve into the photophysical mechanism of carrier dynamics. The fitting results reveal that the TA average decay lifetime of the (HtrzT)PbI_3_(1.0)/FAPbI_3_ perovskite film is approximately twice (9762 vs. 4861 ps) as long as that of the pristine α-FAPbI_3_ film (Fig. 3i and Table S4). The fast component (τ_1_) arises from carrier trapping and non-radiative recombination at surface or grain boundary defects, while the slow component (τ_2_) corresponds to bulk-phase carrier recombination [61]. The longer τ_1_ (696.7 vs. 441.5 ps) and reduced Rel_1_% (4.41% vs. 6.92%) for (HtrzT)PbI_3_(1.0)/FAPbI_3_ compared to pristine α-FAPbI_3_ indicate a reduction in non-radiative recombination pathways associated with defect states. Moreover, (HtrzT)PbI_3_(1.0)/FAPbI_3_ exhibits a significantly prolonged bulk-phase carrier lifetime (10,180 ps), approximately twice that of pure FAPbI_3_ (5189 ps). This enhancement can be attributed to the synergistic effects of enlarged grain size (Fig. S17) and effective defect passivation by LD (HtrzT)PbI_3_ perovskite, which significantly suppresses the fast decay component while prolonging the slow component lifetime, which contributes to attaining an enhancing performance of device.

### Charge Transport Property Characterization

**Fig. 4:**
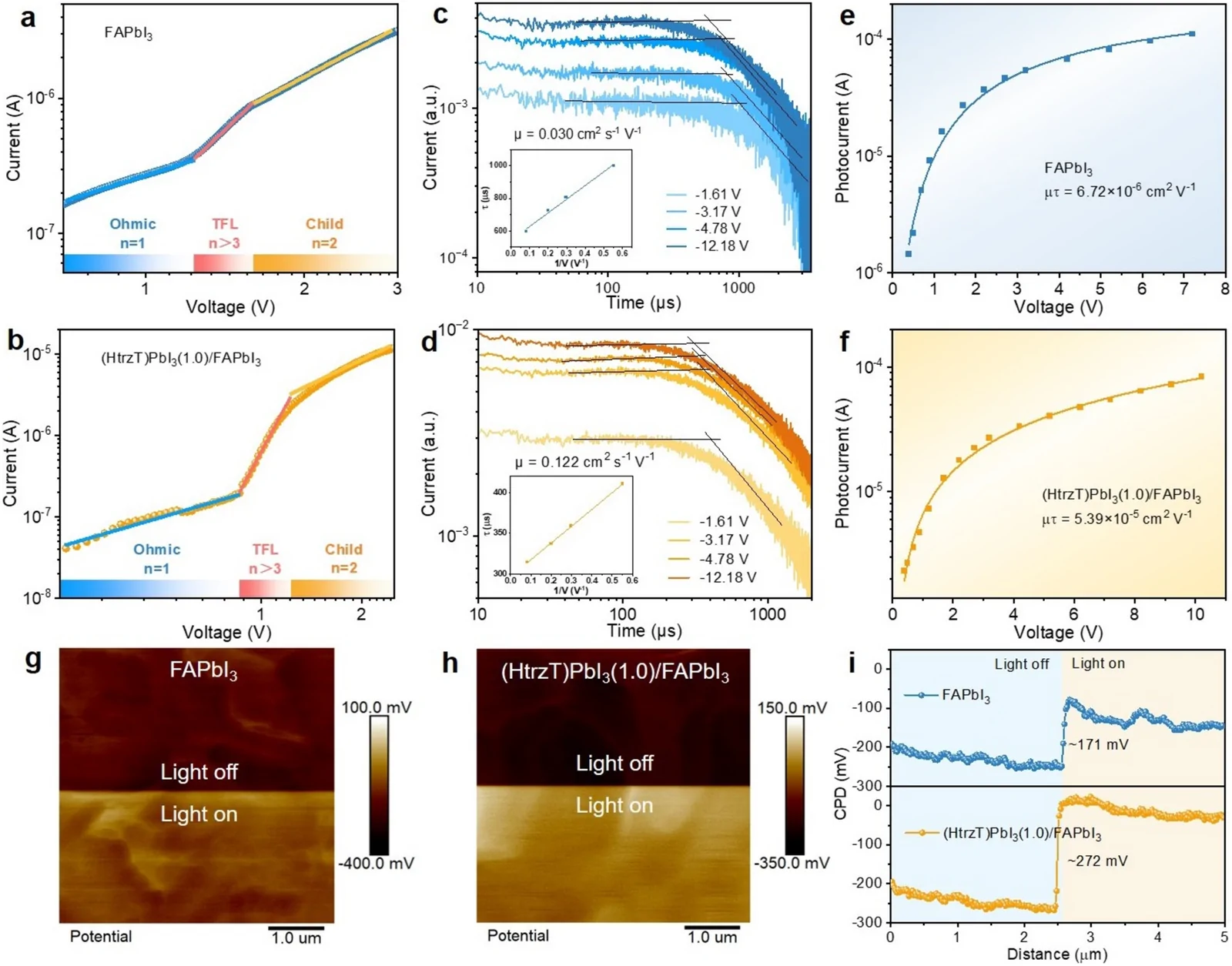
Carrier transport properties and Kelvin probe force microscopy. Dark current–voltage characteristics of the **a** FAPbI_3_ and **b** (HtrzT)PbI_3_(1.0)/FAPbI_3_ perovskite devices measured using the SCLC method. Time-of-flight measurement for the **c** FAPbI_3_ and **d** (HtrzT)PbI_3_(1.0)/FAPbI_3_ perovskite films under 405-nm laser. Photo-electronic measurement for the **e** α-FAPbI_3_ and **f** (HtrzT)PbI_3_(1.0)/FAPbI_3_ perovskite films of photoconductivity curves. The corresponding CPD images of **g** α-FAPbI_3_ and **h** (HtrzT)PbI_3_(1.0)/FAPbI_3_ perovskite films, at light off and light on regions. **i** CPD variety at light on/off, in the **g, h** regions

In order to characterize the trap density and carrier mobility of the perovskite films, the space charge limited current (SCLC) measurement was carried out. The linear Ohmic region, trap-filled limited (TFL) region, and trap-free Child's region can be clearly observed from low voltage to high voltage in SCLC curves (Fig. 4a, b). The trap-filled limit voltage (V_TFL_) represents the kink point voltage between the ohmic region and the TFL region, from which it can be computed that the trap density of the (HtrzT)PbI_3_(1.0)/FAPbI_3_ perovskite film (9.23 × 10^11^ cm^−3^) is marginally smaller than that of the pristine α-FAPbI_3_ film (1.84 × 10^12^ cm^−3^). By fitting the child region of the SCLC curve with the Mott–Gurney law [10], the carrier mobility of the (HtrzT)PbI_3_(1.0)/FAPbI_3_ perovskite film was quantified as 1.44 cm^2^ s^−1^ V^−1^, which is sevenfold superior to that of the pristine film (0.21 cm^2^ s^−1^ V^−1^). The enhanced carrier mobility was further validated using the time-of-flight (ToF) method, in which the measured transit time was fitted to the reciprocal of the applied bias. The results indicate that the carrier mobility of the pristine α-FAPbI_3_ film is calculated as 0.030 cm^2^ s^−1^ V^−1^, while the (HtrzT)PbI_3_(1.0)/FAPbI_3_ perovskite film shows an increased value of 0.122 cm^2^ s^−1^ V^−1^ (Fig. 4c, d), consistent with the trend observed in SCLC measurement. The carrier mobility-lifetime (µτ) product is a crucial parameter for evaluating the transport capability of carriers, which can be measured by the photoconductivity method and analyzed through fitting the photocurrent curve with the modified Hecht equation [44]. The µτ product value of the pristine α-FAPbI_3_ film was recorded at 6.72 × 10^–6^ cm^2^ V^−1^, while in the (HtrzT)PbI_3_(1.0)/FAPbI_3_ film, the µτ product value escalated by approximately one order of magnitude to 5.39 × 10^–5^ cm^2^ V^−1^ (Fig. 4e, f). These enhancements can be attributed to the introduction of trace (HtrzT)PbI_3_, which improves the phase purity of α-FAPbI_3_ and reduces defect density, therefore leading to a prolonged carrier lifetime and an improved charge carrier transport performance. Notably, the electrical conductivity of (HtrzT)PbI_3_ reaches up to 1.27 × 10^–5^ S m^−1^ (as shown in Fig. S18), which is higher than that of some previously reported LD Pb-based perovskite materials ((4,4'-bpy)PbI_4_: 7.4 × 10^–7^ S m^−1^; 5-AQPbI_4_: 5.95 × 10^–8^ S m^−1^; (F-PEA)_2_PbI_4_: 3.28 × 10^–10^ S m^−1^) [34, 62, 63]. When combined with the type-I heterojunction band structure formed between (HtrzT)PbI_3_ and FAPbI_3_, Fowler–Nordheim tunneling plays a key role in charge transport under an applied electric field [64]. Owing to the good electrical conductivity of (HtrzT)PbI_3_, this characteristic ensures efficient and rapid charge transfer across the (HtrzT)PbI_3_/FAPbI_3_ heterojunction interface.

To further confirm that the (HtrzT)PbI_3_(1.0)/FAPbI_3_ perovskite film demonstrates superior optoelectronic properties compared to the pristine α-FAPbI_3_ film, the photo-induced spatial charge distribution of the perovskite films was analyzed using the Kelvin probe force microscope (KPFM) measurement. As illustrated in the contact potential difference (CPD) mapping images (Fig. 4g, h) and the corresponding linear profiles (Fig. 4i), the surface potential of the (HtrzT)PbI_3_(1.0)/FAPbI_3_ perovskite film exhibits a significant positive shift with a change of approximately 272 mV following illumination, exceeding that of the pristine α-FAPbI_3_ film (~ 171 mV). The enhanced surface photovoltage signal indicates an increased hole concentration on the surface, suggesting the improved carrier separation efficiency in the (HtrzT)PbI_3_(1.0)/FAPbI_3_ perovskite film. Furthermore, CPD under illumination is higher than that in the dark, confirming the n-type semiconductor behavior of perovskite films [65]. This observation is consistent with the energy band structure results. The n-type characteristic likely arises from donor-type defects, such as iodine vacancies [66, 67]. Notably, the (HtrzT)PbI_3_(1.0)/FAPbI_3_ film exhibits a lower average CPD (− 233 mV) compared to pristine FAPbI_3_ film (− 222 mV) in the dark state, indicating a higher work function and weaker n-type behavior, which is conducive to reducing the concentration of n-type self-doping [68]. The reduction in n-type character can be attributed to the passivation of iodine vacancies in FAPbI_3_ by (HtrzT)PbI_3_.

### X-Ray Detector Performance Characterization

**Fig. 5:**
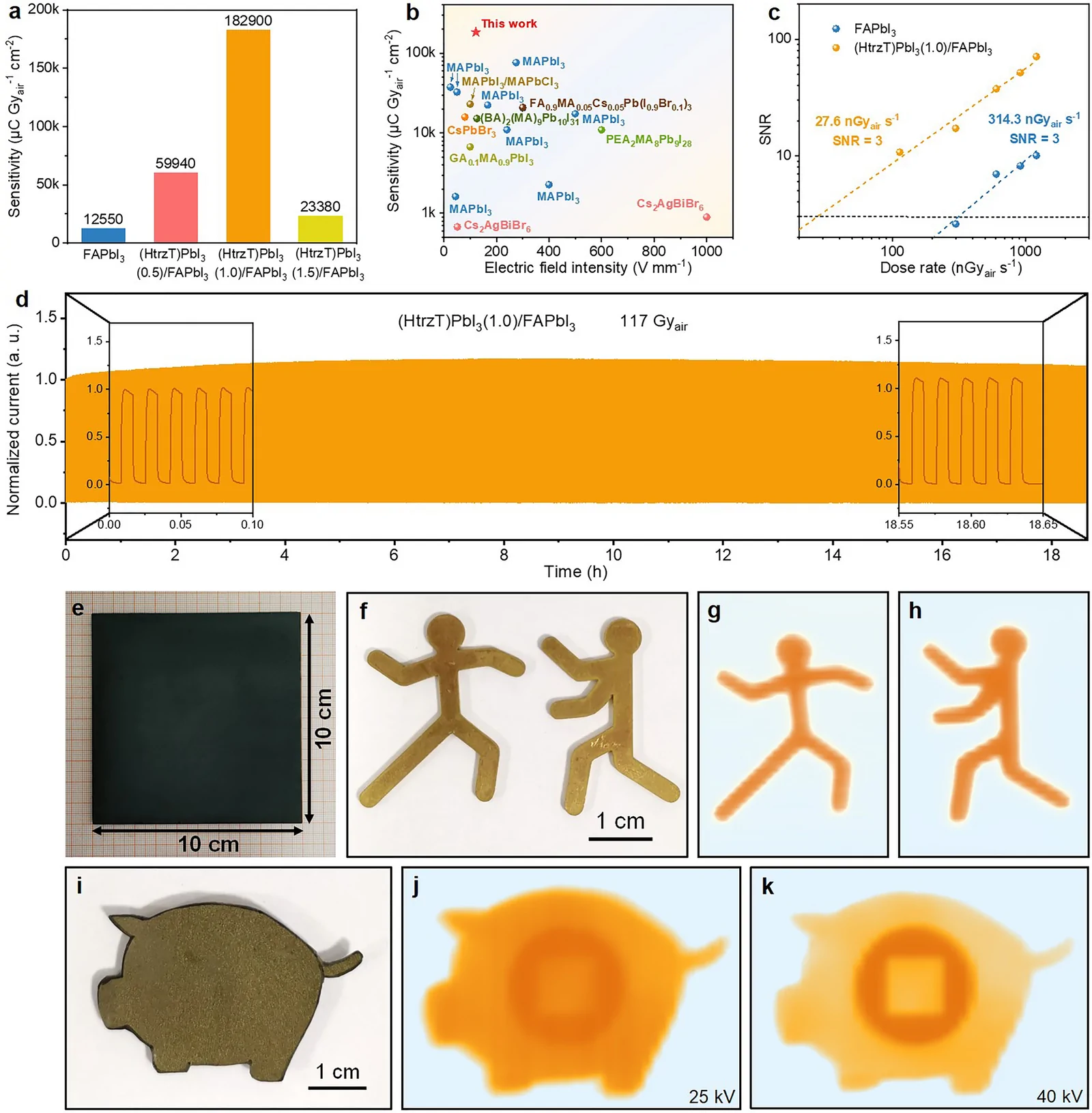
Sensitivity, detection limit, stability and X-ray images for X-ray detectors. **a** Sensitivity of α-FAPbI_3_ and (HtrzT)PbI_3_/FAPbI_3_ perovskite X-ray detectors. **b** Summary of the reported sensitivity to electric field intensity for X-ray detectors based on perovskite thick films by slurry blading. **c** Signal-to-noise ratio (SNR) of the detectors under low dose rates. The black dashed line represents a SNR of 3. **d** X-ray response stability test of the detectors based on the (HtrzT)PbI_3_(1.0)/FAPbI_3_ perovskite film under − 2 V bias. **e** Large-area (HtrzT)PbI_3_(1.0)/FAPbI_3_ perovskite films of 10 cm × 10 cm. **f** Optical images and **g, h** X-ray images of characters performing “kung fu” under a X-ray tube voltage of 40kV. **i** Optical images of the “pig” with a copper coin behind it. X-ray images for “pig” and the copper coin under the X-ray tube voltage of **j** 25 kV and **k** 40 kV

For the X-ray detection performance, the pristine α-FAPbI_3_ and (HtrzT)PbI_3_/FAPbI_3_ perovskite films with the FTO/APTES/PVK/Au device structure were measured in a metal vacuum chamber. As illustrated in Fig. S19a, the (HtrzT)PbI_3_(1.0)/FAPbI_3_ device exhibits a pronounced response to X-ray under an applied bias voltage of − 5 V, with detectable signals across a broad dose rate range from 631.2 to 60.26 µGy_air_ s^−1^. Under an X-ray irradiation dose rate of 631.2 µGy_air_ s^−1^, the net photogenerated current density for (HtrzT)PbI_3_(1.0)/FAPbI_3_ device reaches 117 µA cm^−2^, approximately 13-fold higher than that of the device composed of the pristine α-FAPbI_3_ film (about 9 µA cm^−2^). The response current of the devices exhibits a linear dependence on the incident X-ray dose rate, arising from the proportional generation of photogenerated carriers within the perovskite active layer and their efficient extraction under an applied bias voltage. Figure S19b summarizes the response current densities of devices with varying amounts of (HtrzT)PbI_3_ addition corresponding to different X-ray dose rates, and the slope obtained through linear fitting is employed to calculate the detection sensitivity of devices, with the results compiled in Fig. 5a. With the increasing amounts of (HtrzT)PbI_3_ addition from (HtrzT)PbI_3_(0.5)/FAPbI_3_ to (HtrzT)PbI_3_(1.0)/FAPbI_3_, the sensitivity improved from an initial value of 12,550 μC Gy_air_^−1^ cm^−2^ to 182,900 μC Gy_air_^−1^ cm^−2^ and subsequently decreases to 23,380 μC Gy_air_^−1^ cm^−2^ for (HtrzT)PbI_3_(1.5)/FAPbI_3_. Notably, the sensitivity of the (HtrzT)PbI_3_(1.0)/FAPbI_3_ perovskite device was significantly elevated by approximately 15-fold compared to that of the pristine α-FAPbI_3_ film device. Furthermore, the present sensitivity surpasses the reported values for most of X-ray detectors based on perovskite polycrystalline thick films by blade coating at a low electric field (Fig. 5b) [13, 51, 69–76]. Such remarkable improvement in charge collection capability is ascribed to the fact that (HtrzT)PbI_3_ addition not only reduces the residual stress and formation energy of the perovskites, but also improves the phase purity of α-FAPbI_3_. Consequently, sensitivity of the (HtrzT)PbI_3_(1.0)/FAPbI_3_ device is significantly improved. Utilizing the X-ray attenuation coefficient of α-FAPbI_3_ derived from the photon cross-section database (Fig. S20) and assuming 100% charge collection efficiency, the theoretical sensitivity of (HtrzT)PbI_3_(1.0)/FAPbI_3_ is calculated (Formula 9) to be 269 µC Gy_air_^−1^ cm^−2^, resulting in a gain factor of 680 relative to the experimental value of 182,900 µC Gy_air_^−1^ cm^−2^. This remarkable enhancement, arising from both the superior μτ product and electric field-driven carrier reinjection, establishes exceptional X-ray detection performance [77]. According to the IUPAC standard, a signal-to-noise ratio (SNR) value of 3 is regarded as a detection limit. With X-ray irradiation dose rate gradually decreasing from 1207.2 nGy_air_ s^−1^ to 113.7 nGy_air_ s^−1^, the detector based on the (HtrzT)PbI_3_(1.0)/FAPbI_3_ device exhibits a significant response at -3 V bias voltage (Fig. S21a), whereas the pristine α-FAPbI_3_ detector fails to exhibit a discernible photocurrent response at the low dose rate of 113.7 nGy_air_ s^−1^ (Fig. S21b). Figure 5c presents the relationship between the dose rate and SNR, signifying that the (HtrzT)PbI_3_(1.0)/FAPbI_3_ perovskite detector exhibits a detection limit of 27.6 nGy_air_ s^−1^. This detection limit is much lower than that of the pristine α-FAPbI_3_ detector (314.3 nGy_air_ s^−1^) and approximately 200 times lower than that for current medical diagnosis (5.5 µGy_air_ s^−1^). The low detection limit arises from the significantly reduced noise level of the (HtrzT)PbI_3_(1.0)/FAPbI_3_ detector compared to pristine α-FAPbI_3_ (Fig. S22). The (HtrzT)PbI_3_(1.0)/FAPbI_3_ detector exhibits a response time comparable to the pristine FAPbI_3_ detector under X-ray (Fig. S23), confirming that introducing (HtrzT)PbI_3_ LD perovskite does not introduce substantial charge traps to impede carrier transport. Continuous radiation stability constitutes a crucial parameter for an X-ray detector in practical applications. The response of the unpackaged (HtrzT)PbI_3_(1.0)/FAPbI_3_ X-ray detector was conducted under on–off X-ray radiation with a high radiation dose (117 Gy_air_), which is approximately 1.17 × 10^6^ times of the dose required for a single X-ray chest radiography (0.1 mGy_air_ per time) [78]. During the test period exceeding 18 h, both dark current and photocurrent of the device remained stable (Fig. 5d), demonstrating the outstanding radiation stability and reversible switching response of (HtrzT)PbI_3_(1.0)/FAPbI_3_ detector.

There is a substantial and growing demand for large-area detectors in various X-ray imaging applications, such as medical imaging and security screening. The blade coating technology offers a facile and efficient approach for fabricating uniform and large-area perovskite films. A larger area (HtrzT)PbI_3_(1.0)/FAPbI_3_ perovskite film with a size of 10 cm × 10 cm was also fabricated (Fig. 5e). The PL mappings of the film were tested at 9 distinct positions. The results indicated that the (HtrzT)PbI_3_(1.0)/FAPbI_3_ perovskite film with hot-pressing exhibited more uniform PL intensity (Fig. S24b) across different positions, compared to the film without hot-pressing (Fig. S24a). To explore the imaging potential of the (HtrzT)PbI_3_(1.0)/FAPbI_3_ X-ray detector, a single-pixel detector was employed for X–Y scanning imaging, with an integration time of 0.1 s per pixel. Under the irradiation of X-ray with a dose rate of 631.2 μGy_air_ s^−1^, well-defined X-ray images for characters performing various action of “kung fu” could be acquired (Fig. 5f–h). As shown in Figs. 5i and S25, the copper coin placed behind the copper-containing epoxy resin “pig” is invisible in visible light. At a lower X-ray tube voltage of 25 kV with X-ray energy range of approximately 15–25 keV, the “pig” outline exhibits clear spatial resolution, while the copper coin pattern remains poorly defined due to strong X-ray absorption for copper-containing epoxy resin “pig” (Fig. 5j). Relatively, the patterns of both the “pig” and the copper coin were clearly manifested under a higher X-ray tube voltage of 40 kV and X-ray energy range around 15–40 keV (Fig. 5k). The difference in image contrast can be attributed to the energy-dependent X-ray attenuation coefficients of the materials. The high-Z copper exhibits stronger absorption than the copper-containing epoxy resin "pig" at higher energies, and increasing the tube voltage to 40 kV enhances penetration through both materials, enabling simultaneous visualization of their structural features. These results suggest that the X-ray detector exhibits excellent imaging capability and application potential.

## Conclusions

In conclusion, we have realized stable and high-performance FA-based perovskite X-ray detectors through regulating the lattice stress of perovskites with matched lattice. The LD (HtrzT)PbI_3_ perovskite demonstrates a robust chemical synergy with α-FAPbI_3_, helping to mitigate lattice expansion and thus effectively suppress phase transitions in α-FAPbI_3_. This phenomenon provides support for a stable Pb-I octahedral framework in the FAPbI_3_ perovskite, which induces the growth of stable α-phase perovskite. Combined effects of defect passivation and phase purity improvement, the (HtrzT)PbI_3_(1.0)/FAPbI_3_ perovskite film demonstrates a significant twofold increase in carrier lifetime and a nearly one-order-of-magnitude enhancement of the μτ product. As a result, the electrical performance of (HtrzT)PbI_3_/FAPbI_3_ perovskite film was significantly enhanced. The (HtrzT)PbI_3_(1.0)/FAPbI_3_ X-ray detector achieves an excellent sensitivity of 1.83 × 10^5^ μC Gy_air_^−1^ cm^−2^, with a detection limit as low as 27.6 nGy_air_ s^−1^. Additionally, this detector demonstrated superior X-ray radiation stability and could tolerate a radiation dose of 117 Gy_air_. The study provides a novel idea for addressing the lattice strain issue in α-FAPbI_3_ perovskites, and lay the foundation for the future development of high-performance and stable α-FAPbI_3_ perovskite X-ray detectors.

## Supplementary Information

Below is the link to the electronic supplementary material.

Supplementary file1 (DOCX 5534 KB)
