# Supplementary material for: Lattice Anchoring Stabilizes α-FAPbI3 Perovskite for High-Performance X-Ray Detectors
Source: Nanomicro Lett. 2025 Jul 29;18:14. doi: 10.1007/s40820-025-01856-4 (PMC12307842; doi:10.1007/s40820-025-01856-4)
Supplement: Supplementary file 1 — Supplementary file1 (DOCX 5534 KB) [file 40820_2025_1856_MOESM1_ESM.docx]

Supporting Information for

**Lattice Anchoring Stabilizes α-FAPbI_3_ Perovskite for High-Performance X-Ray Detectors**

Yu-Hua Huang^1^, Su-Yan Zou^1^, Cong-Yi Sheng^1^, Yu-Chuang Fang^1^, Xu-Dong Wang^1,^ *, Wei Wei^1^, Wen-Guang Li^1^ and Dai-Bin Kuang^1,^ *

^1^ Key Laboratory of Bioinorganic and Synthetic Chemistry of Ministry of Education, LIFM, GBRCE for Functional Molecular Engineering, School of Chemistry, IGCME, Sun Yat-Sen University, Guangzhou 510275, P. R. China

*Corresponding authors. E-mail: [wangxd26@mail.sysu.edu.cn](mailto:wangxd26@mail.sysu.edu.cn) (Xu-Dong Wang); [kuangdb@mail.sysu.edu.cn](mailto:kuangdb@mail.sysu.edu.cn) (Dai-Bin Kuang)

**Supplementary Figures**


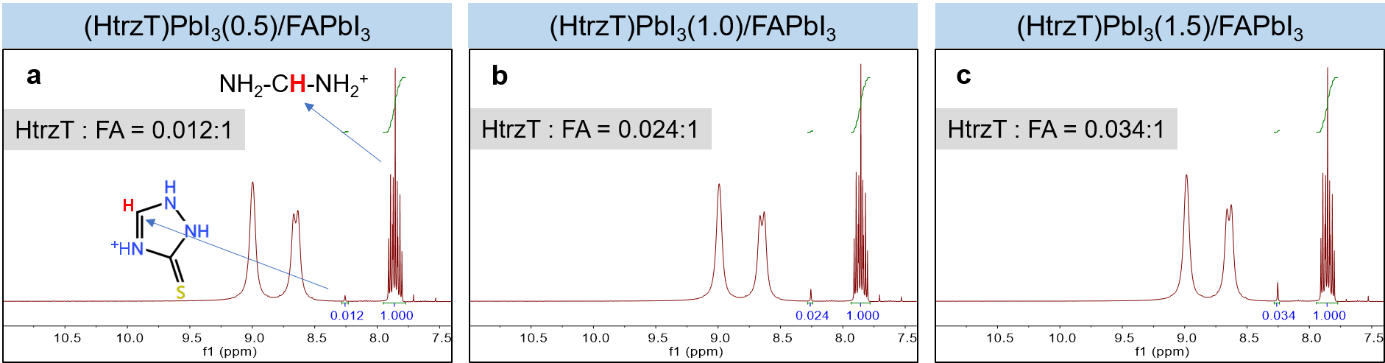


**Fig. S1** ^1^H NMR spectrum of the (**a**) (HtrzT)PbI_3_(0.5)/FAPbI_3_, (**b**) (HtrzT)PbI_3_(1.0)/FAPbI_3_, (**c**) (HtrzT)PbI_3_(1.5)/FAPbI_3_


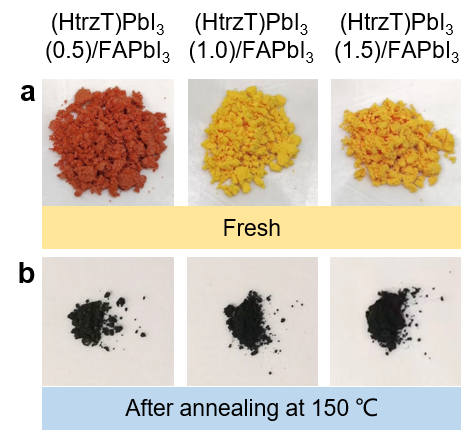


**Fig. S2** Optical photographs of (HtrzT)PbI_3_/FAPbI_3_ perovskite microcrystals (**a**) before and (**b**) after annealing at 150 °C


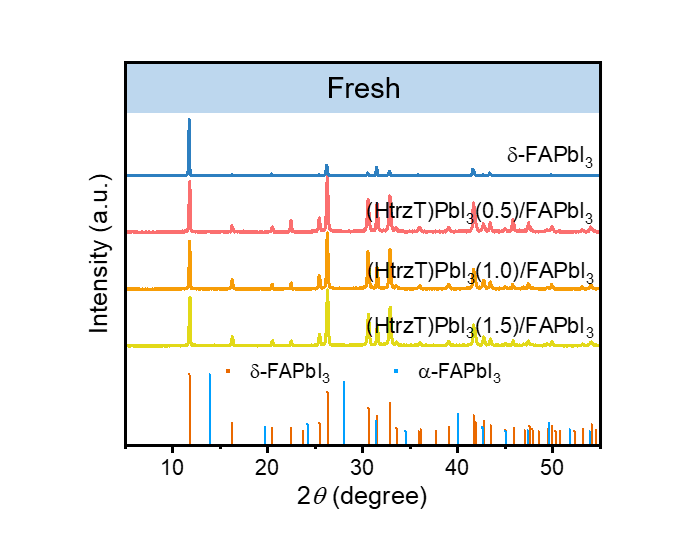


**Fig. S3** The XRD patterns of freshly prepared FAPbI_3_ and (HtrzT)PbI_3_/FAPbI_3_ microcrystals


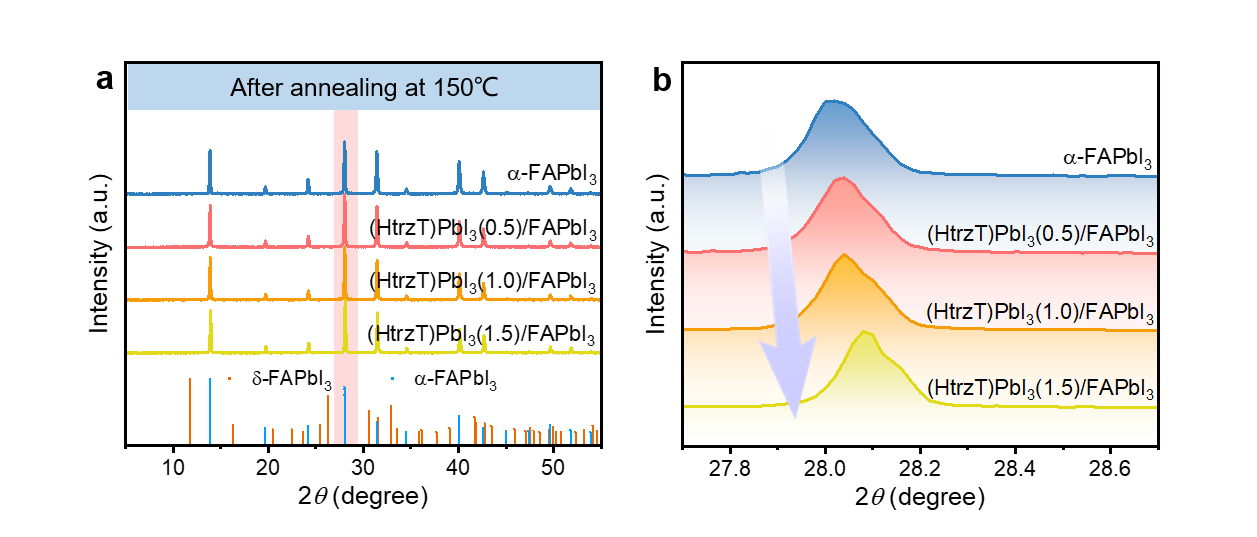


Fig. S4 (a) The XRD patterns of FAPbI_3_ and (HtrzT)PbI_3_/FAPbI_3_ microcrystals after annealing at 150 °C. (b) Magnified (002) diffraction peaks in the region shaded in red in (a)


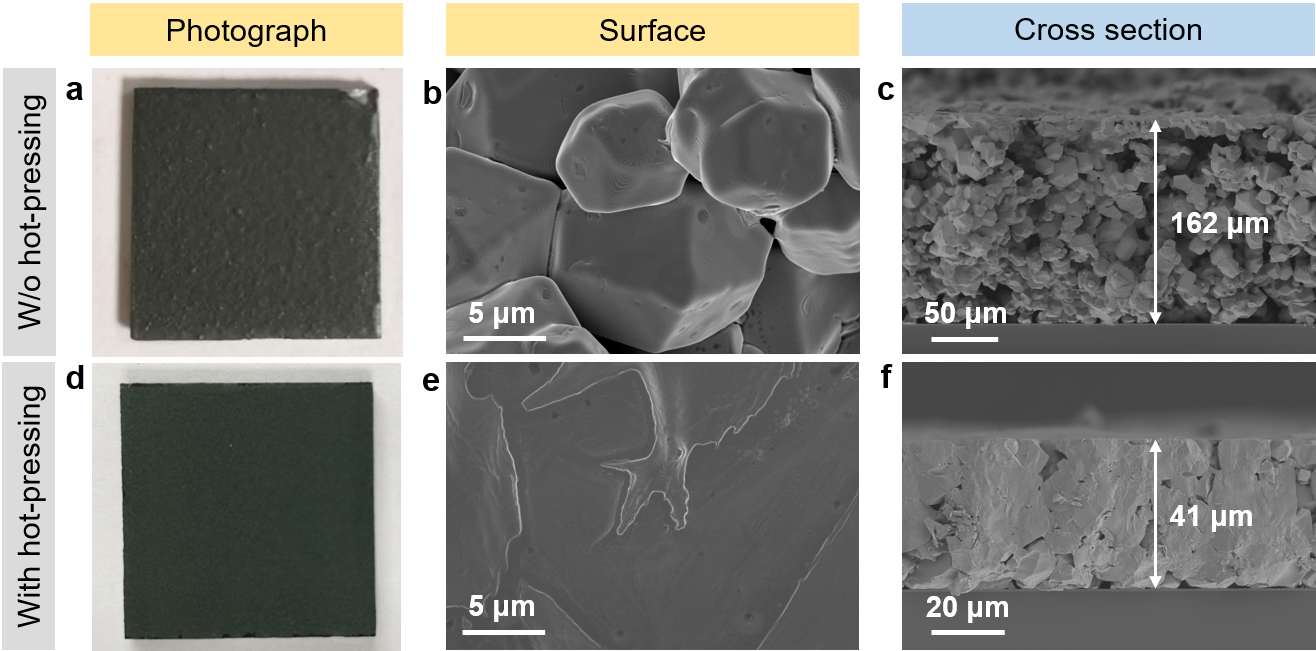


**Fig. S5** The photograph of the (HtrzT)PbI_3_(1.0)/FAPbI_3_ films (**a**) w/o and (**d**) with hot-pressing. The SEM images of the (HtrzT)PbI_3_(1.0)/FAPbI_3_ films (**b**) w/o and (**e**) with hot-pressing. Cross-sectional SEM images of the (HtrzT)PbI_3_(1.0)/FAPbI_3_ films (**c**) w/o and (**f**) with hot-pressing


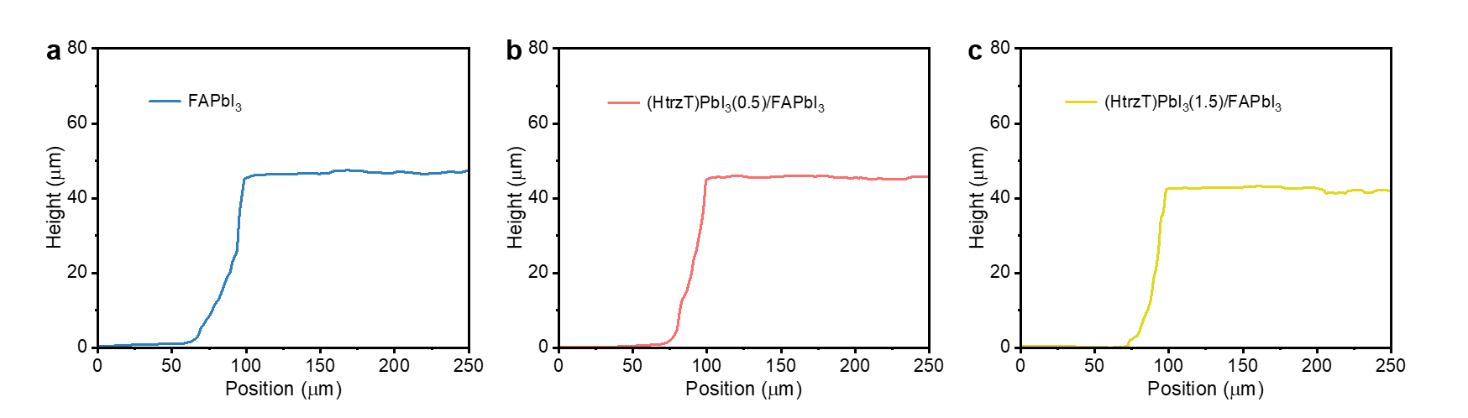


**Fig. S6** The thickness profile curves of the (**a**) FAPbI_3_, (**b**) (HtrzT)PbI_3_(0.5)/FAPbI_3_ and (**c**) (HtrzT)PbI_3_(1.5)/FAPbI_3_ films with hot-pressing


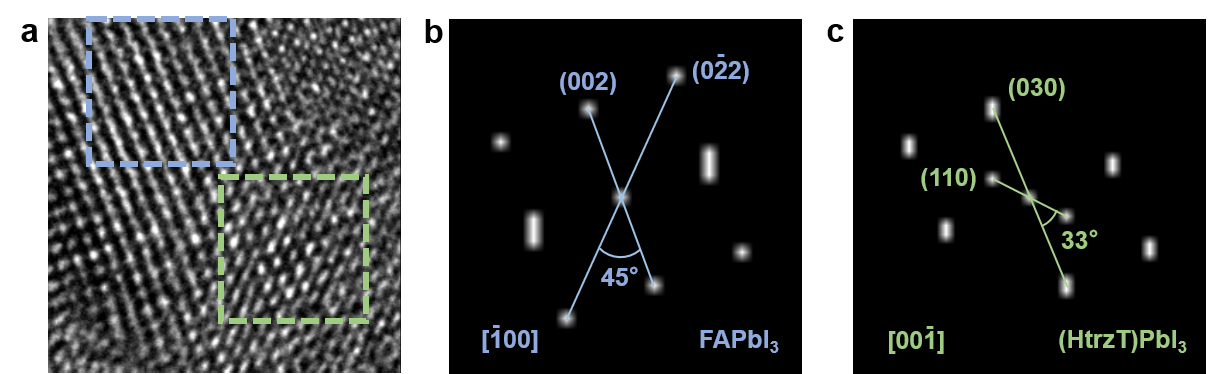


**Fig. S7** (**a**) The TEM image of (HtrzT)PbI_3_(1.0)/FAPbI_3_, are the same as that in Fig. 1c. Images in (**b**) and (**c**) are fast Fourier transform (FFT) of the blue and green dashed square in **a**, corresponding to the FAPbI_3_ and (HtrzT)PbI_3_ perovskites, respectively


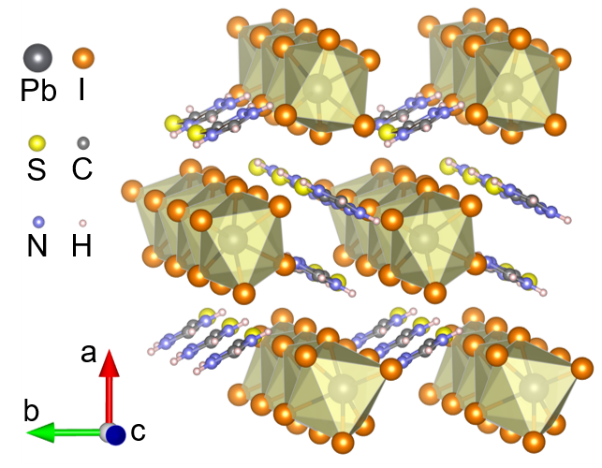


**Fig. S8** The crystal structure of 1D (HtrzT)PbI_3_ perovskite


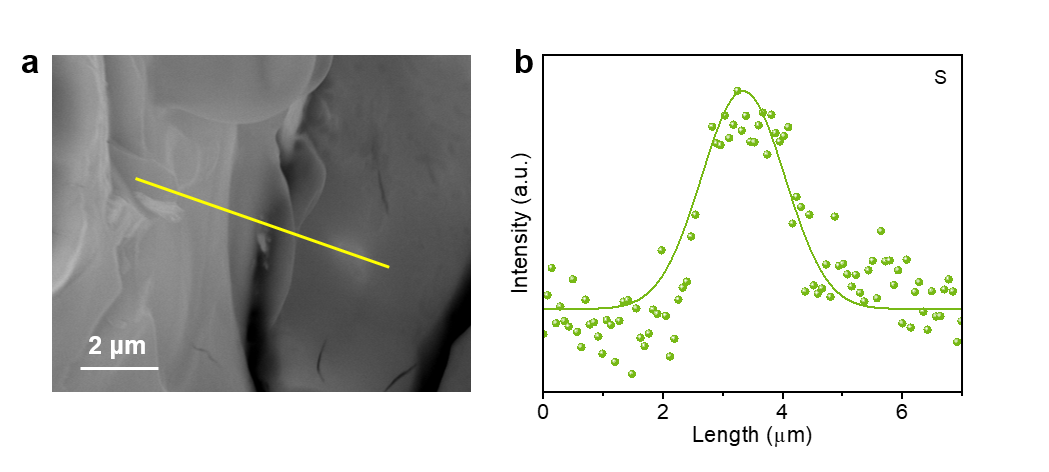


**Fig. S9** (**a**) SEM image and (**b**) the corresponding EDS line scan spectrum (S element) of (HtrzT)PbI_3_(1.0)/FAPbI_3_ perovskite film


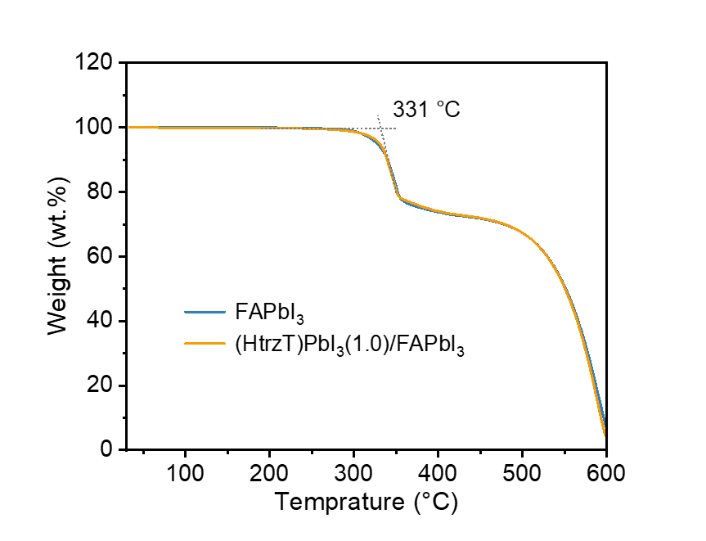


**Fig. S10** TGA curves of FAPbI_3_ and (HtrzT)PbI_3_(1.0)/FAPbI_3_


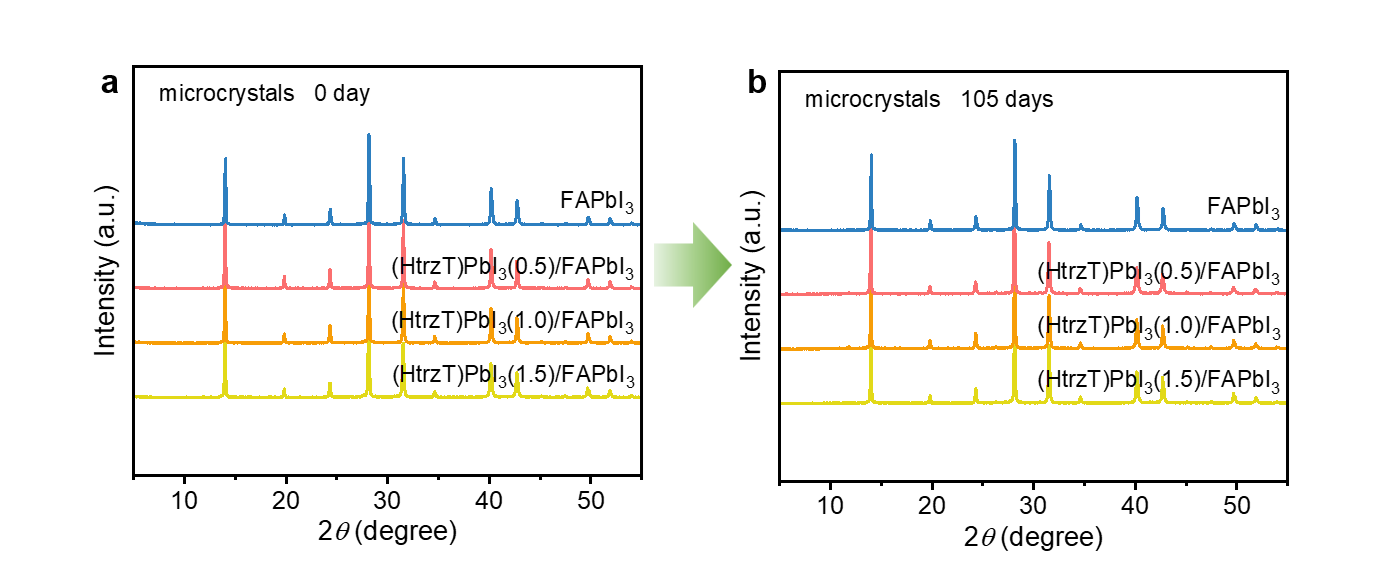


**Fig. S11** The XRD patterns of FAPbI_3_ and (HtrzT)PbI_3_/FAPbI_3_ perovskite microcrystals after being stored in a nitrogen atmosphere for (**a**) 0 day and (**b**) 105 days


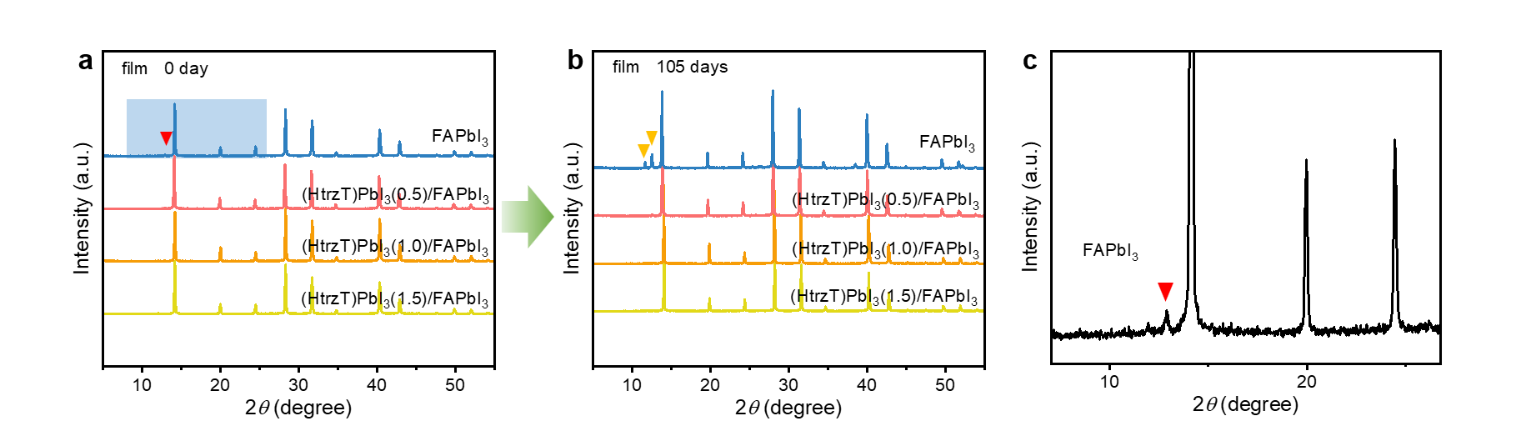


**Fig. S12** The XRD patterns of FAPbI_3_ and (HtrzT)PbI_3_/FAPbI_3_ perovskite films after being stored in a nitrogen atmosphere for (**a**) 0 day and (**b**) 105 days. (**c**) Diffraction peaks of FAPbI_3_ film in the region shaded in blue in (**a**)


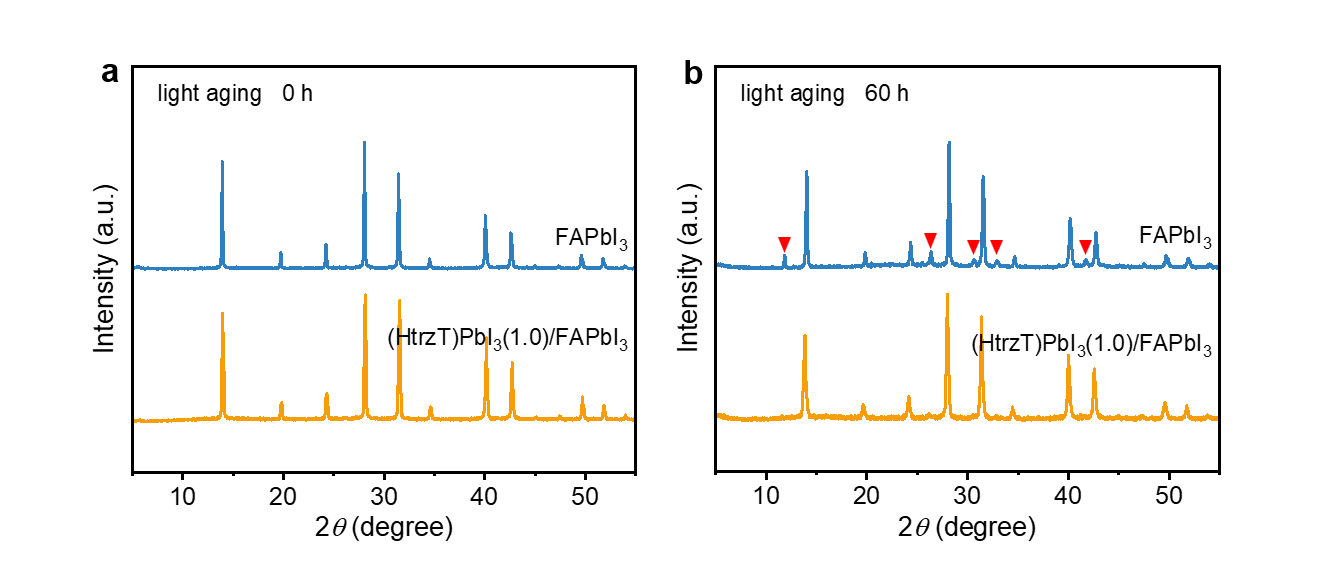


**Fig. S13** The XRD patterns of FAPbI_3_ and (HtrzT)PbI_3_/FAPbI_3_ perovskite microcrystals (**a**) before and (**b**) after light aging (100 mW cm^-2^) for 60 hours


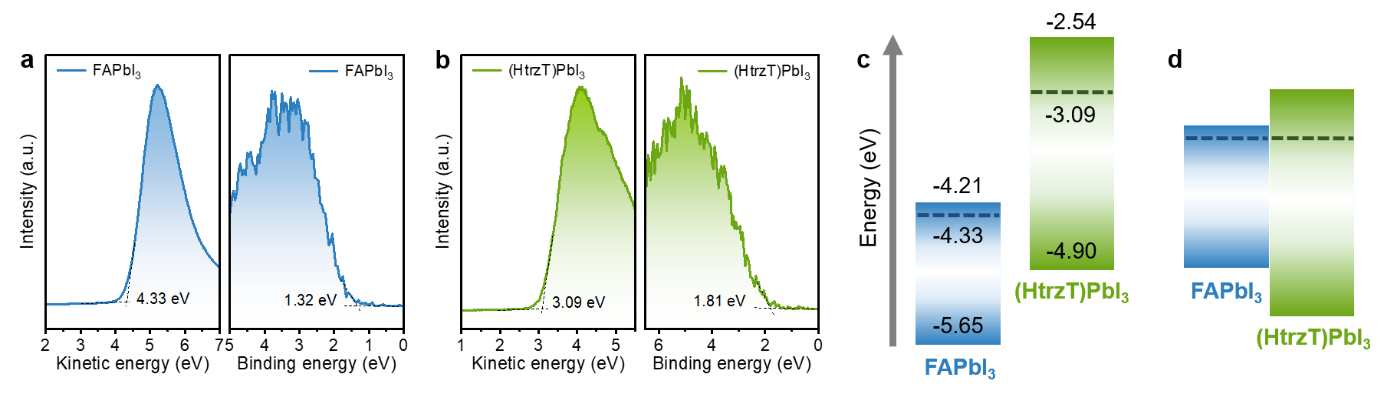


**Fig. S14** Secondary electron cutoff XPS spectra and valence spectra of (**a**) FAPbI_3_ and (**b**) (HtrzT)PbI_3_. Schematic illustration of the band structure of (**c**) FAPbI_3_ and (HtrzT)PbI_3_, and (**d**) (HtrzT)PbI_3_/FAPbI_3_ perovskite.


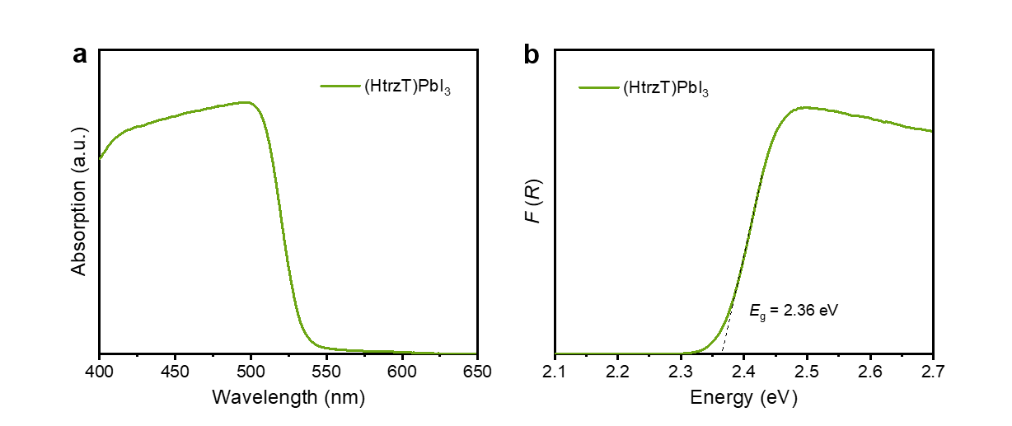


**Fig. S15** (**a**) Absorption spectrum of (HtrzT)PbI_3_. (**b**) Kubelka-Munk plot for determining the band gap of the (HtrzT)PbI_3_


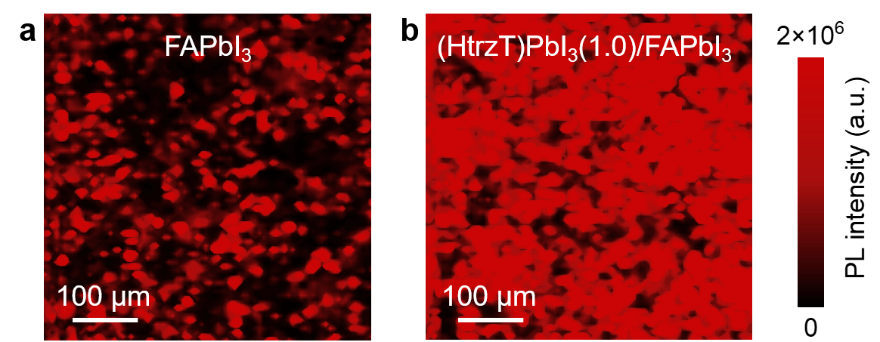


**Fig. S16** PL mappings of (**a**) α-FAPbI_3_ and (**b**) (HtrzT)PbI_3_(1.0)/FAPbI_3_ perovskite


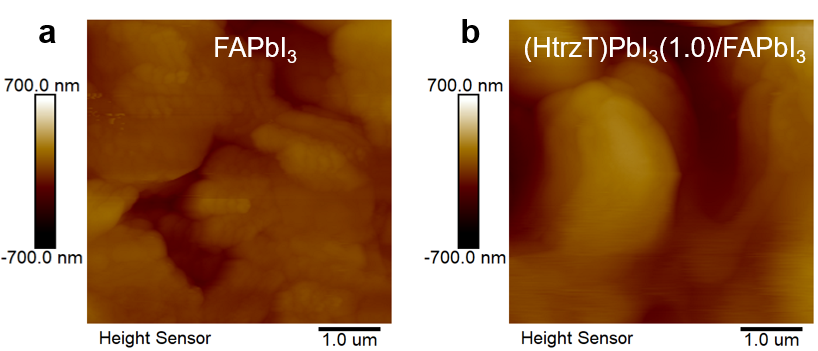


**Fig. S17** AFM topography of (**a**) FAPbI_3_ and (**b**) (HtrzT)PbI_3_(1.0)/FAPbI_3_ films


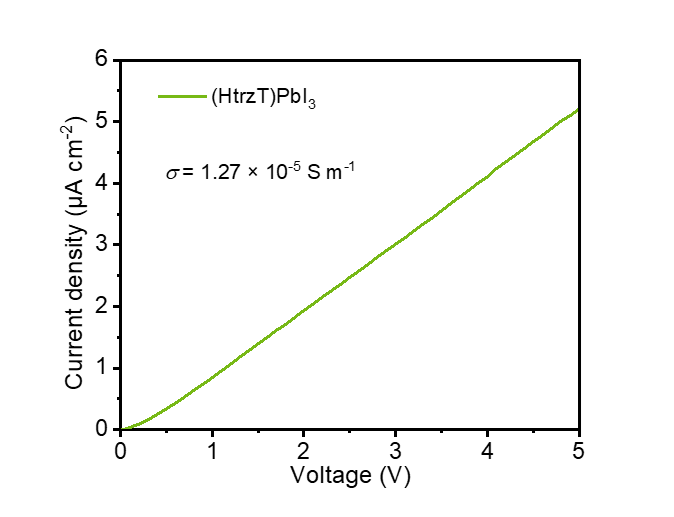


**Fig. S18** The I-V curve of the (HtrzT)PbI_3_ device in the dark


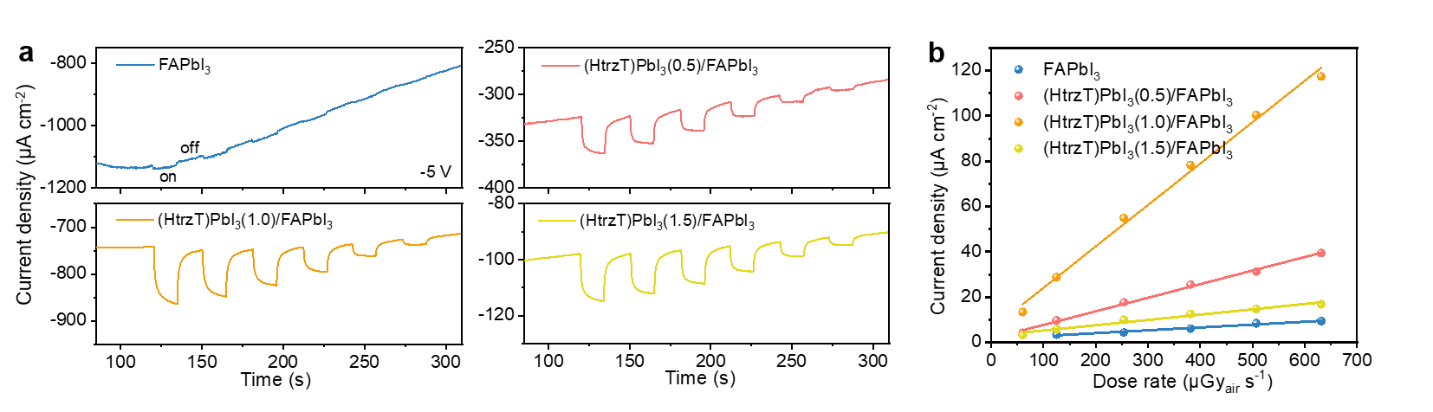


**Fig. S19** (**a**) X-ray responses of the FAPbI_3_ and (HtrzT)PbI_3_/FAPbI_3_ films under a series of dose rates from 631.2 to 60.26 µGy_air_ s^-1^. (**b**) The X-ray induced current density versus dose rate of the FAPbI_3_ and (HtrzT)PbI_3_/FAPbI_3_ perovskite detectors


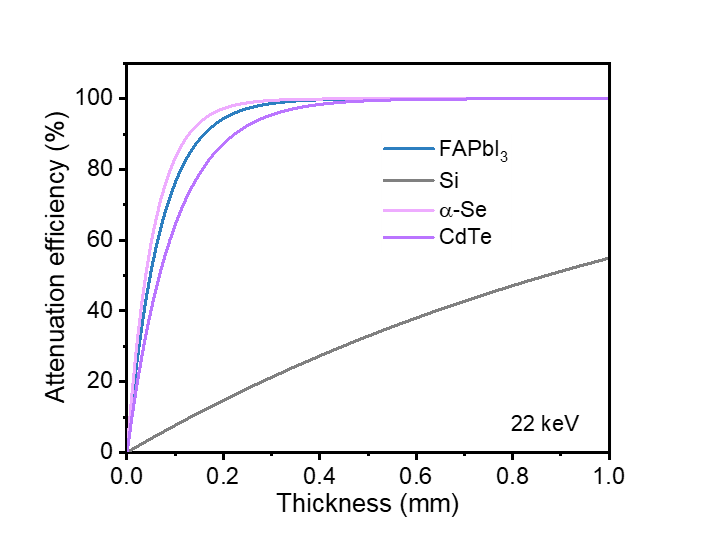


**Fig. S20** Attenuation efficiencies as a function of material thickness for Si, α-Se, CdTe, and FAPbI_3_ at 22 keV X-ray photons


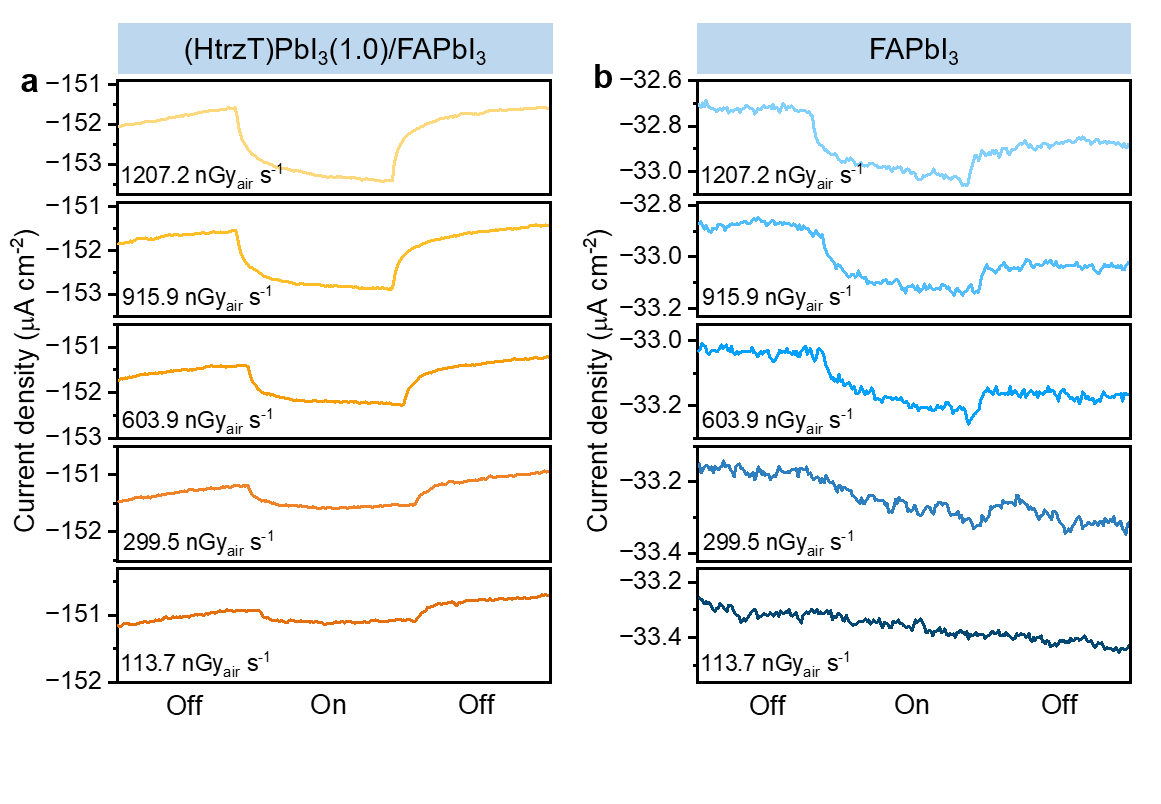


**Fig. S21** The X-ray responses of the device based on the (**a**) (HtrzT)PbI_3_(1.0)/FAPbI_3_ and (**b**) FAPbI_3_ films under on-off X-ray irradiation with various dose rates at -3 V bias


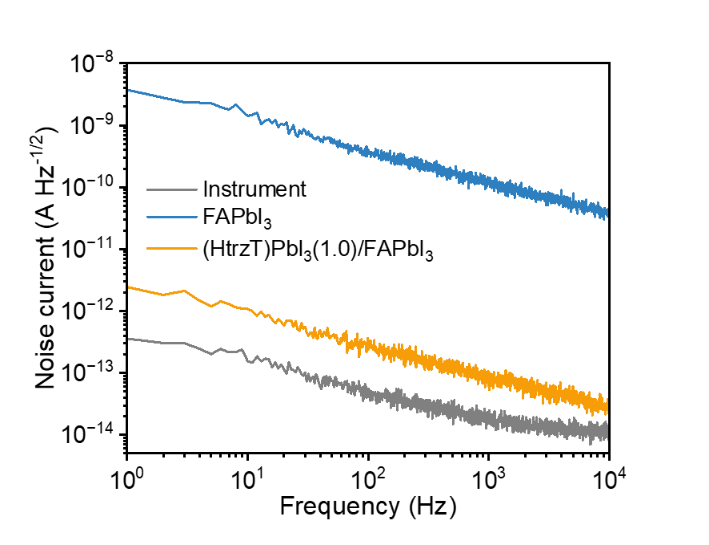


**Fig. S22** The noise currents of the FAPbI_3_ and (HtrzT)PbI_3_(1.0)/FAPbI_3_ devices


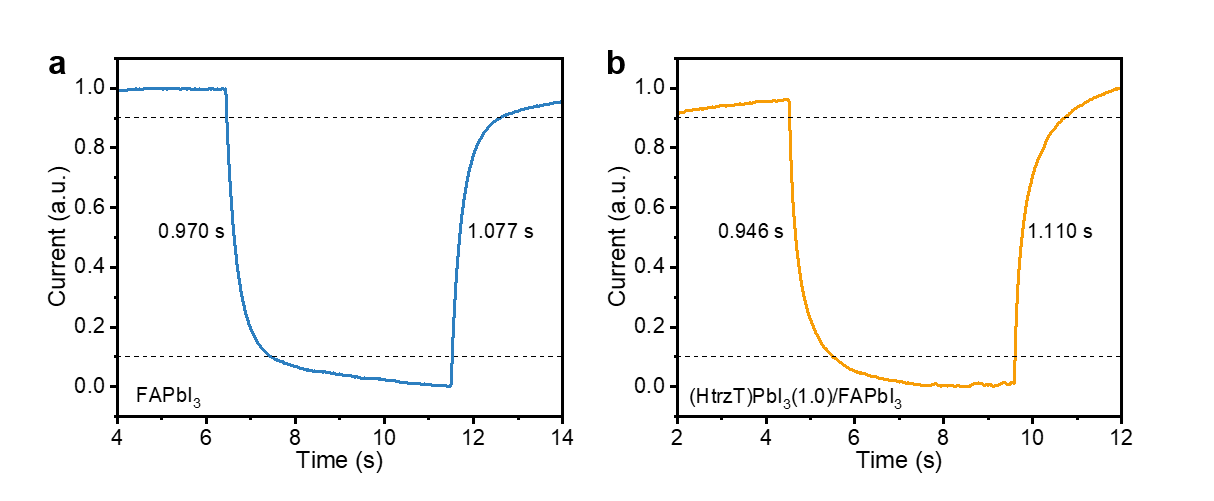


**Fig. S23** The response time of (**a**) FAPbI_3_ and (**b**) (HtrzT)PbI_3_(1.0)/FAPbI_3_ devices under X-ray irradiation


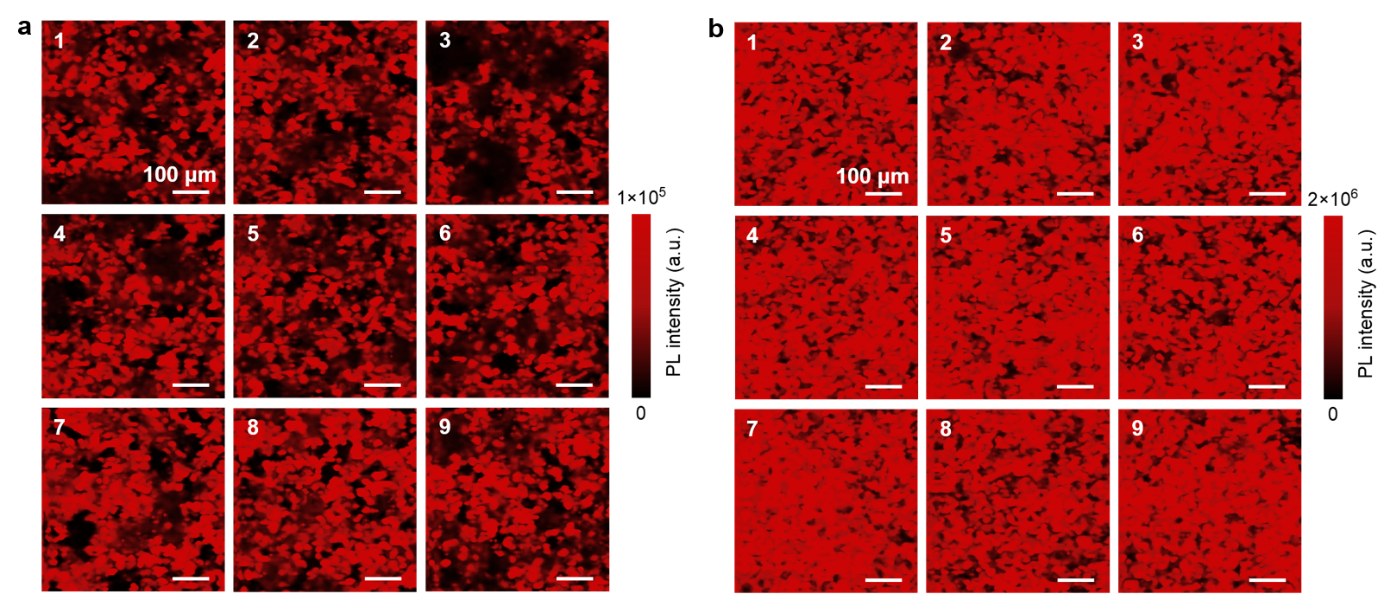


**Fig. S24** Sampling at nine points on an area of 5 cm × 5 cm for PL mappings of (HtrzT)PbI_3_(1.0)/FAPbI_3_ perovskite films (**a**) without and (**b**) with hot-pressing. The scale bars are 100 µm


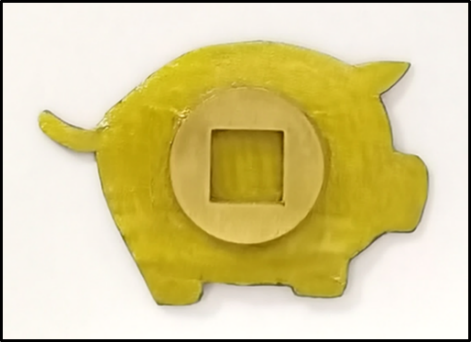


**Fig. S25** Backside optical image of “pig” with a copper coin for X-ray imaging

**Supplementary Tables**

**Table S1** The composition of organic precursor solution for the FAPbI_3_ and (HtrzT)PbI_3_/FAPbI_3_ perovskite films

| Sample | Mole fractions of HtrzT:FA | HtrzT/mmol | FAAc/mmol | HI/mL | H_3_PO_2_/μL |
| --- | --- | --- | --- | --- | --- |
| FAPbI_3_ | 0:1 | 0 | 5 | 0.5 | 20 |
| (HtrzT)PbI_3_(0.5)/FAPbI_3_ | 0.5:1 | 2.5 | 5 | 1.25 | 50 |
| (HtrzT)PbI_3_(1.0)/FAPbI_3_ | 1:1 | 5 | 5 | 2.5 | 100 |
| (HtrzT)PbI_3_(1.5)/FAPbI_3_ | 1.5:1 | 7.5 | 5 | 3.75 | 150 |

Table S2 Crystal Data for the (HtrzT)PbI_3_ single crystal

| Compound | (HtrzT)PbI_3_ |
| --- | --- |
| Empirical formula | C_2_H_4_I_3_N_3_PbS |
| Formula weight | 690.03 |
| Crystal system | orthorhombic |
| Space group | *Pna*2_1_ |
| Unit cell dimensions | *a* = 13.9364(5) Å,  *b* = 8.9355(3) Å  *c* = 8.7176(3) Å  *α* = 90^o^, *β* = 90^o^, *γ* = 90^o^ |
| Volume | 1085.59(7) Å^3^ |
| Z | 4 |
| Density (calculated) | 4.222 g cm^-3^ |
| F(000) | 1176.0 |
| Index ranges | -19 ≤ h ≤ 19, -12 ≤ k ≤ 12,  -11 ≤ l ≤ 11 |
| Independent reflections | 2813 [R_int_ = 0.0595, R_sigma_ = 0.0431] |
| Data/restraints/parameters | 2813/1/92 |
| Goodness-of-fit on F^2^ | 1.031 |
| Final R indexes [I>=2σ (I)] | R_1_ = 0.0284, wR_2_ = 0.0537 |
| Largest diff. peak/hole | 1.14/-1.14 e^.^Å^-3^ |

Table S3 Lifetime of TRPL for the FAPbI_3_ and (HtrzT)PbI_3_/FAPbI_3_ perovskite films

| Sample | τ_1_/ns | Rel_1_% | τ_2_/ns | Rel_2_% | τ_avg_/ns |
| --- | --- | --- | --- | --- | --- |
| FAPbI_3_ | 32.87 | 72.4 | 409.6 | 27.6 | 137 |
| (HtrzT)PbI_3_(0.5)/FAPbI_3_ | 100.2 | 56.77 | 617.6 | 43.23 | 324 |
| (HtrzT)PbI_3_(1.0)/FAPbI_3_ | 126.2 | 52.22 | 704.6 | 47.78 | 403 |
| (HtrzT)PbI_3_(1.5)/FAPbI_3_ | 203.7 | 39.35 | 1197.2 | 60.65 | 806 |

Table S4 Lifetime of TA for the FAPbI_3_ and (HtrzT)PbI_3_(1.0)/FAPbI_3_ films

| Sample | τ_1_/ps | Rel_1_% | τ_2_/ps | Rel_2_% | τ_avg_/ps |
| --- | --- | --- | --- | --- | --- |
| FAPbI_3_ | 441.5 | 6.92 | 5189 | 93.08 | 4861 |
| (HtrzT)PbI_3_(1.0)/FAPbI_3_ | 696.7 | 4.41 | 10180 | 95.59 | 9762 |
